# Development of a unique crosslinked glycosaminoglycan for soft tissue repair: Treatment of interstitial cystitis/bladder pain syndrome

**DOI:** 10.1371/journal.pone.0317790

**Published:** 2025-01-24

**Authors:** Richard W. Heidebrecht, Thomas H. Jozefiak, Harrison C. Shain, Eugene M. Skrabut, Debra Saunders, Nataliya Smith, Rheal A. Towner, Robert Hurst

**Affiliations:** 1 Glycologix, Inc., 100 Cummings Center, Beverly, Massachusetts, United States of America; 2 Advanced Magnetic Resonance Center, Oklahoma Medical Research Foundation, Oklahoma City, Oklahoma, United States of America; 3 University of Prince Edward Island, Charlottetown, Prince Edward Island, Canada; National Cancer Institute at Frederick, UNITED STATES OF AMERICA

## Abstract

Chemical modification of naturally derived glycosaminoglycans (GAGs) expands their potential utility for applications in soft tissue repair and regenerative medicine. Here we report the preparation of a novel crosslinked chondroitin sulfate (~200 to 2000 kilodaltons) that is both soluble in aqueous solution and microfilterable. We refer to these materials as “SuperGAGs.” One can further conjugate these materials with diverse capture agents to further modify polymer properties and add new capabilities. A representative material (GLX-100) demonstrated durable restoration of bladder impermeability in a gold standard animal model of Interstitial Cystitis/Bladder Pain Syndrome (IC/BPS). Histologic examination of the animal bladders treated with a GLX-100 SuperGAG conjugated to biotin as a reporter demonstrated that the residence time of GLX-100 is superior to chondroitin sulfate (a product that is currently used for clinical treatment of patients with IC/BPS). As expected, this novel crosslinked GAG biopolymer was restricted to the luminal surface of the bladder wall. In this communication we describe a simple and versatile synthesis of a crosslinked glycosaminoglycan (GAG) biopolymer for soft tissue repair. Chondroitin sulfate (~12 kD) was crosslinked to form a water soluble and microfilterable polymer with approximately 200 to 2000 kD molecular weight. The synthesis presented here allows for control of molecular weight while avoiding formation of an extended block gel. Moreover, the procedure enables further chemical modification of the SuperGAG through the selection of a capture agent. A set of agents have been used, demonstrating the preparation of a family of SuperGAGs with diverse capabilities. We can optimize polymer properties, adjust adherence to various tissues, add reporters, and engage the biochemistry of surrounding tissues with peptides and other bioactives.

## Introduction

Interstitial cystitis/bladder pain syndrome (IC/BPS) is a chronic bladder condition characterized by urinary urgency and frequency with pain of more than 6 months duration [1]. The disorder can be crippling [2]. The prevalence of IC/BPS is difficult to assess due to heterogeneity within the disorder, nonobjective diagnostic criteria, and a lack of suitable biomarkers. Estimates of prevalence in the United States are 3.3 to 7.9 million [3,4]. The etiology is not definitively known, but multiple pathophysiologic features have been proposed [3]. Several investigators have identified a dysfunction of the bladder urothelium [5,6]. Most significant is increased bladder permeability due to the loss of the luminal GAG layer [5,7–9]. This GAG layer is the first line of defense against urinary solutes in contact with the bladder wall [10–13]. This bladder permeability dysfunction allows penetration by urinary potassium (and other solutes) which leads to sensory nerve upregulation, pain, and urinary frequency/urgency [14,15]. A compromised urothelium can lead to a chronic inflammatory response that prevents the GAG layer from being reestablished [16]. Some patients develop pelvic and lower gastrointestinal symptoms due to pelvic nerve cross-talk [17].

Therapeutic options for patients are limited with only 2 drugs approved within the United States–intravesical dimethylsulfoxide (DMSO) and oral sodium pentosanpolysulfate (PPS) [18]. PPS is an oral GAG replacement therapy that has been implicated in maculopathy [19]. A variety of intravesical GAG replacement therapies (e.g., chondroitin sulfate (CS), hyaluronic acid (HA) and/or heparin) have shown limited therapeutic success but none have been approved by the FDA [20]. Compelling animal studies suggest that current clinical implementation of GAG replenishment therapy may not be achieving its full potential. GAG biopolymers instilled intravesically have been shown to restore impermeability. The administered GAG biopolymers bind preferentially to bladder urothelium lacking the GAG layer or luminal cell layer [21], restoring bladder impermeability while inhibiting recruitment of inflammatory cells in the bladder wall [22].

There are three limitations to GAG replenishment therapy as it is currently practiced. First, is the heterogeneity of the disorder with potentially multiple etiologies, not all of which may respond to GAG replacement therapy [23]. Second, is the nature of the GAGs that are administered. The natural GAG layer is comprised of branched proteoglycans that form a dense, deep impermeable GAG layer. Currently administered GAGs are linear often lower molecular weight biopolymers that may not mimic the proteoglycans responsible for the impermeability of the natural GAG layer. Third, the residence “dwell” time of instilled GAG replacement on the bladder wall may not be long enough to maintain coverage between treatments. If the bladder urothelium loses impermeability between instillations, those interventions are less likely to interrupt the inflammatory cascade encountered in some patients.

In this communication we describe the preparation of a specific SuperGAG material (GLX-100) that restores a damaged GAG-deficient bladder membrane to its normal impermeable state. In a mouse model of bladder permeability, we show that GLX-100 provides both enhanced efficacy and superior adherence to the luminal surface of the bladder when compared with chondroitin sulfate (a standard of care outside the US). Enhanced residence time on the bladder surface will maintain impermeability between instillations. We also show that GLX-100 displays excellent biocompatibility, satisfying preclinical safety standards. A phase 1b clinical trial in IC/BPS patients is ongoing.

## Materials and methods

### Synthesis of SuperGAG polymers

**Fig 1:**
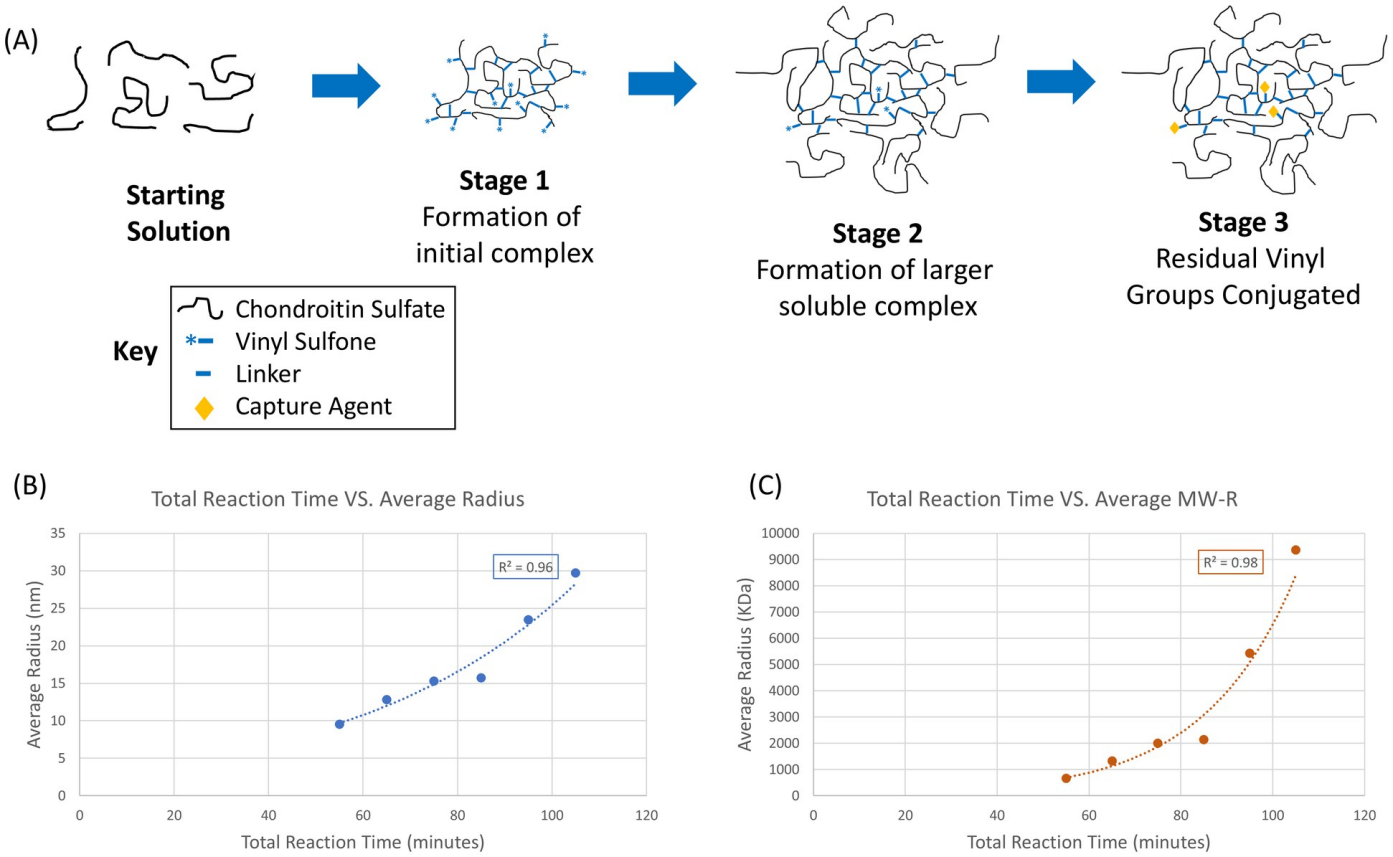
Graphical view of SuperGAG polymer synthesis with control of the molecular weight of the product. (A) Graphical depiction of the preparation of SuperGAG polymer. The longer the crosslinking reaction proceeds, the higher the molecular weight of the material generated. (B) Total reaction time vs average Dynamic Light Scattering (DLS) hydrodynamic radius (n = 2). (C) Total reaction time vs average MW-R (n = 2).

A SuperGAG with enhanced performance in animal models of permeable bladder was synthesized via a method similar to one previously described [24]. The critical step is a multistage addition of the chondroitin sulfate polymer to the crosslinking reaction as depicted in Fig 1A. The reaction between chondroitin sulfate and divinylsulfone is initiated with base (Stage 1). After 15 minutes, an additional and larger portion of chondroitin sulfate is added, and the reaction is allowed to run for a specific second time period (Stage 2, typically 65 minutes). There are opportunities to adjust the properties of the polymer in this multistage reaction sequence. As expected, longer stage 2 reaction times lead to higher average molecular weight for the resulting polymer (Fig 1B and 1C). One interesting observation in the previously reported synthetic method is that the resulting polymer retains a small amount of pendant vinylsulfone after purification (depicted in Stage 2). These residual vinylsulfones do not degrade quickly over time in isolated samples. Quenching of this situationally reactive functional group is probably desirable when used to treat a disease in humans [25]. This feature of the reaction can be used to our advantage to further functionalize the polymer with any number of capture agents (Stage 3, vide infra).

General Procedure for SuperGAG Derivatives: A solution of sodium chloride (93.1 mg,) water (9.56 g) and chondroitin sulfate (345 mg) was prepared. This homogenous solution was treated with divinylsulfone (154 μL, 1.54 μmol) followed by aqueous sodium hydroxide (1.06 mL, 1N NaOH) and the resulting yellow solution was stirred magnetically. After 15 minutes another portion of chondroitin sulfate (1.04 g) was added. The resulting suspension was mixed using a Vortex Genie mixer until the polymer was completely dissolved. After 65 additional minutes the requisite capture agent (1.54 μmol) was added and the reaction stirred for an additional 15 minutes. The pH of the solution was adjusted to ~7 with 1N aqueous hydrochloric acid, then diluted to a volume of ~40 mL with Dulbecco’s Phosphate Buffered Saline (DPBS.) This solution was subjected to 0.2-micron filtration and purified via a 100 kD membrane via tangential flow filtration. The retentate was exchanged against 8 volumes of DPBS then 5 volumes of water. The retentate solution was again filtered (0.2 micron) and lyophilized to provide the target polymer as a white to light yellow solid.

### Animal studies

All animal studies were approved by the Oklahoma Medical Research Foundation (OMRF) Institutional Animal Care and Use Committee (IACUC). Adult female, homozygous URO-MCP-1 mice (20-25g) were used for the study. For all in vivo procedures, mice were anesthetized with isoflurane (1.5–2.0% with 800–1,000 mL oxygen/min). On day 0, lipopolysaccharide (LPS) (1 μg in 100 μL DPBS) was infused into the urinary bladder via an intravesical catheter. A lubricated (lidocaine jelly, 2%) sterile catheter (24-gauge x ¾ inch) was used for transurethral catheterization of each animal. LPS was instilled in the bladder and retained for 10 minutes. LPS was then removed from the bladder using abdominal pressure and followed by 3 saline flushes (100 μL per flush). This dilute LPS concentration is insufficient to induce permeability in wild-type mice, but sufficient to induce permeability in URO-MCP-1 mice. Saline-treated URO-MCP-1 mice were used as controls. Bladder hyper-permeability was assessed by MRI at 3- and 5-days following exposure to LPS (n = 5–8 for each group). Sham catheterized controls were administered saline (100 μL) instead of LPS. The next day (Day 1) animals were treated with chondroitin sulfate (CS, 20 mg/mL in saline; 100 μL) GLX-100 (20 mg/mL in saline; 100 μL) or saline (100 μL) and permeability was assessed 3 days after LPS treatment (Day 3). To assess the stability of GLX-100 on the bladder surface, mice were treated with a biotinylated GLX-100 and terminated at day 1, 5 or 10.

### Bladder permeability measurements

Bladder permeability (in vivo) was determined directly using magnetic resonance imaging (MRI) as described previously [24,26]. Briefly, MRI experiments were conducted on a 7 Tesla 30 cm-bore Bruker Biospec MRI system on anesthetized mice (see above), that were restrained in a cradle, which was inserted into the MRI scanner. All animals had their body temperatures maintained at 37° C with a water bath driven heating pad placed under the animal in the MRI cradle. Respiratory rates were also monitored for the entire duration of the MRI scan. The MRI contrast agent Gd-DTPA was instilled by intravesical infusion into the bladder. A RARE (rapid acquisition with refocused echoes) T1 sequence was used for the contrast-enhanced MRI study. Signal intensity was compared before and 7 minutes after the Gd-DTPA instillation. Permeability was assessed by calculating a percent increase in MRI signal intensity just outside the bladder wall from MRI images (5 regions-of-interest (ROI) per animal). MRI signal intensities and T1 values were measured from ROIs that visually expressed label within images as previously described [24,27]. ROIs were taken in bladder walls from images and T1 maps, along with corresponding regions in saline animal datasets.

### Histology and immunohistochemistry (IHC)

Mice are euthanized by CO_2_ in an approved delivery system, followed by cervical dislocation, before the bladders are extracted. This method is consistent with the recommendations of the American Veterinary Medical Association (AVMA) Guidelines on Euthanasia. The removed bladders were preserved in 10% neutral buffered formalin and processed routinely by embedding in paraffin and sectioning (5 μm) onto HistoBond^®^ Plus slides. Sections were deparaffinized, rehydrated, and stained according to standard protocols for hematoxylin and eosin staining. The sections for immunohistochemistry were developed as described previously [24] using the M.O.M^®^ (Mouse on Mouse) ImmPRESS^®^ Polymer kit, Peroxidase. Antigen retrieval (Antigen Unmasking Solution, Citrate-based) was accomplished via 20 minutes in a steamer followed by 30 minutes cooling at room temperature. Sections were treated with a peroxidase blocking reagent Bloxall, followed by M.O.M. IgG blocking reagent to inhibit nonspecific binding. Slides were rinsed in DPBS and then incubated for 5 minutes with M.O.M., and for 20 minutes in 2.5% Normal Horse Serum. Sections for streptavidin horseradish peroxidase (SA-HRP), for the bladders (n = 5/group) where the streptavidin targets the biotin moiety of the biotinylated derivative of the SuperGAG, were processed as above, except they were incubated for 30 minutes at room temperature with ready to use Streptavidin, Peroxidase, R.T.U. Appropriate washes were in PBS. Slides were incubated with Vector^®^ NovaRed^®^ Substrate kit, Peroxidase (HRP) chromogen for visualization. Counterstaining was carried out with Hematoxylin QS Nuclear Counterstain. Appropriate positive and negative tissue controls were used.

IHC labeling was assessed by measuring SA-HRP levels from mouse bladders administered biotinylated SuperGAGs. Five ROIs, captured digitally (20× magnification), were identified in each case. Only areas containing urothelial tissue were analyzed. The number of positive pixels was divided by the total number of pixels (negative and positive) in the analyzed areas as previously described. ROIs were selected by experienced observers in a core lab who were blinded as to the treatment received by the animal. Quantification was achieved using Aperio ImageScope.

### Statistical methods

For efficacy and binding comparisons, both two-way non-parametric Student’s t-tests and two-way ANOVA were applied to raw data to evaluate multiple comparisons between all groups and time points. Percent differences in contrast enhancements and average positivity values for SA-HRP staining were calculated. For efficacy measurements, post- minus pre-contrast MRI signal intensities from Bruker Paravision image analysis software was used to calculate percent changes in MRI signal intensities. For binding levels, APERIO assessed positivity staining analysis was used to calculate binding levels of biotinylated SuperGAGs. Student’s t-tests were applied to determine statistical significance between treated groups vs. control. For both efficacy and binding level measurements, data were analyzed using one way ANOVA, and the differences between means were analyzed using Tukey’s multiple comparisons.

### Safety testing

Biocompatibility testing was performed under Good Laboratory Practice (GLP) at accredited laboratories. The methods and results of safety testing are referenced in “S1 File”.

## Results

### Preparation of SuperGAGs with various capture agents

**Fig 2:**
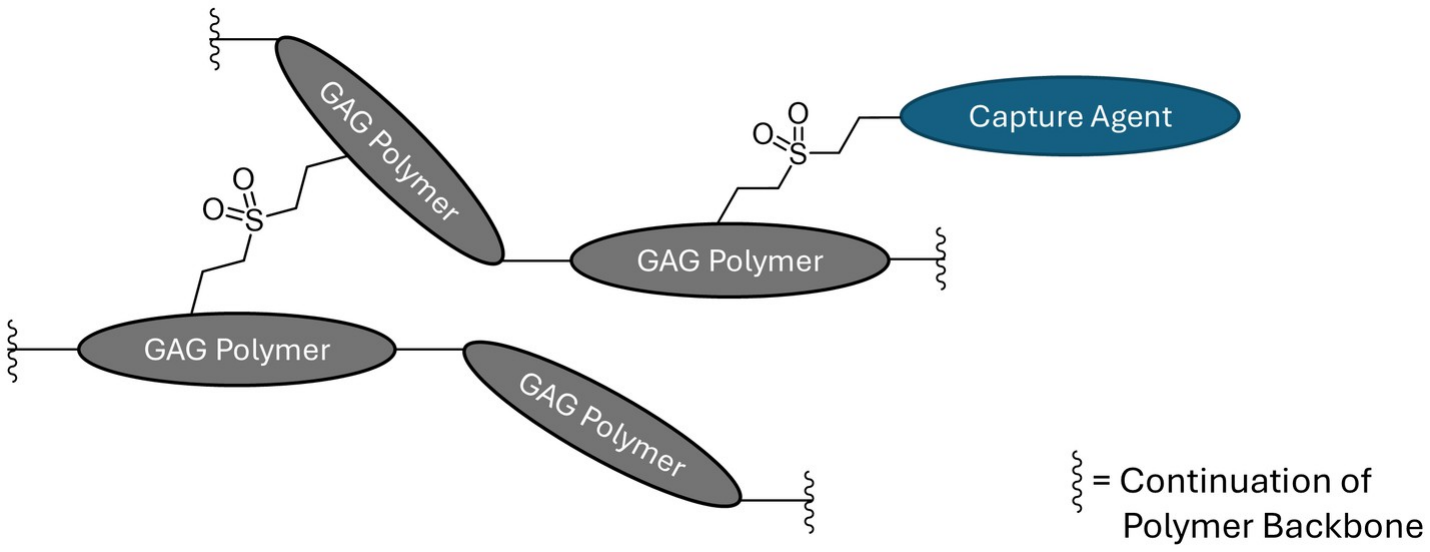
Graphical depiction of crosslinked SuperGAG polymers with capture agents.

Using the general procedure described above we demonstrated the ability to conjugate a diverse set of capture agents. This work is depicted in Fig 2 and summarized in Table 1. Entry 1 represents the parent reaction with no capture agent. This provides the crosslinked material at a molecular weight dialed in by adjusting the time of stage 2. The purpose of the last column in Table 1 is to describe the degree of modification of the resulting SuperGAG by showing the integral for the signal in the 1H NMR spectrum that is diagnostic for each capture agent. In entry 1, there is no capture agent, so the vinyl protons of the vinyl group are shown. Simple amines (entries 2 to 9) and cyclic amines (entries 10 to 14) are excellent capture agents. These agents completely consume all the vinyl sulfone present in the polymer, and entry 5 (capture agent hexylamine, GLX-100) became the lead material for further studies. Diverse amino acids (entries 15 to 21) also undergo conjugation, as well as a dipeptide (entry 22) and those containing sulfur (entries 23 to 26.) Tryptamine (entry 27) and 4-(aminomethyl)benzoic acid (entry 28) add interesting options. Strongly nucleophilic derivatives (entries 29–32) and simple sulfides (33–35) provide conjugated materials. Biotin derivatives (entries 36–37) were conveniently prepared and were used as a reporter in this work (vide infra). Convenient reactive handles were conjugated to SuperGAGs (38–39) opening up options for further derivatization. Very polar examples (40–43) and diamines (44–45) also produce the target materials. In some examples there are multiple potential points of conjugation with the capture agent; we currently assume that the most nucleophilic option is operative.

**Table 1:** SuperGAGs with diverse capture agents.

Entry	Capture Agent	Yield^1^	DLS hydro-dynamic Radius (nanometers)	DLS MW-R^2^ (kDa)	Degree of Modification (^1^H NMR^3^)	Chemical Shift (ppm)
1	None	45%	34	13011	Vinyl CH_2_ = 0.082 H	6.45
2	Methylamine hydrocholoride^4^	44%	23	4899	Methyl group = 0.087 H	2.63
3	Amyl amine	46%	17	2508	Amyl methyl = 0.22 H	1.38
4	1-Butylamine	37%	38	15520	Butyl methyl = 0.28 H	0.96
5	Hexylamine	57%	24	5420	Hexyl methyl = 0.22 H	0.89
6	Heptylamine	49%	25	6003	Heptyl methyl = 0.15 H	0.90
7	n-Methylhexylamine	46%	23	5270	Hexyl methyl = 0.17 H	0.91
8	sec-Butylamine	43%	21	3983	sec-Butyl methyl = 0.16 H	1.02
9	Benzylamine HCl	56%	34	12960	Phenyl = 0.40 H	7.54
10	Pyrrolidine	33%	18	2816	Merged Signal	-
11	4-Methylpiperidine 99%	37%	20	3687	Methyl = 0.15 H	1.03
12	4-Methylpiperidine-4-ol hydrochloride	24%	14	1566	Methyl = 0.10 H	1.33
13	Cyclohexylamine	24%	28	8435	Merged Signal	-
14	Hexamethyleneimine	53%	24	5476	CH_2_ = 0.36	1.73
15	Glycine	43%	20	3569	Merged Signal	-
16	Alanine	43%	19	3123	Methyl = 0.10 H	1.52
17	Serine	34%	20	3642	Merged Signal	-
18	Phenylalanine	46%	21	3979	Phenyl = 0.20 H	7.50
19	Lysine	38%	21	4106	CH_2_ = 0.06	1.50
20	Arginine	41%	20	3645	CH_2_ = 0.06	1.70
21	Beta alanine	33%	17	2491	Methylene = 0.05 H	2.63
22	Glycylphenylalanine	40%	19	3347	Phenyl = 0.16 H	7.36
23	Cysteine	43%	19	3297	Merged Signal	-
24	N-Acetyl-L-cysteine, 98%	48%	21	4340	Methylene = 0.14 H	3.05
25	DL-Homocysteine	59%	19	3331	Methylene = 0.08 H	2.79
26	Glutathione reduced form	37%	18	2874	Methylene = 0.11 H	2.55
27	Tryptamine hydrochloride	21%	31	10544	Aryl = 0.21 H	7.8–7.2
28	4-(Aminomethyl) benzoic acid	44%	20	3949	Aryl = 0.16 H	7.9, 7.55
29	Hydroxylamine hydrochloride	51%	25	6311	Merged Signal	-
30	(2-Hydroxyethyl) hydrazine	23%	20	3856	Methylene = 0.11 H	3.00
31	Acethydrazide	57%	44	23610	Merged Signal	-
32	Valeric acid hydrazide	30%	64	56712	Methyl = 0.12 H	0.92
33	Hexanethiol	71%	47	26985	Methyl = 0.17 H	0.90
34	2-Ethylhexyl thioglycolate	72%	128	289624	Methyl = 0.56 H	0.94
35	2-Mercaptoethanol	44%	18	3081	Methylene = 0.073 H	2.83
36	N-(2-Aminoethyl)biotinamide	42%	18	2929	Methylene = 0.04 H	2.81
37	Biotin-PEG7-amine	48%	23	5209	Methylene = 0.04 H	2.82
38	Propargyl-PeG3-amine	45%	18	2972	Merged Signal	-
39	11-Azido-3,6,9-trioxaundecan-1-amine	42%	17	2689	Merged Signal	-
40	R-3-amino-1, 2-propanediol	28%	30	9369	Merged Signal	-
41	2-2-(2-Methoxyethoxy) ethanamine^5^	33%	17	2517	Merged Signal	-
42	3-Dimethylamino propylamine	43%	25	6407	Dimethylamino = 0.52 H	2.93
43	2-Ethoxyethylamine^5^	15%	16	2326	Ethyl methyl = 0.10 H	1.32
44	1,6-Hexamethydiamine	48%	27	7432	Methylene = 0.35 H	1.46
45	Spermidine	44%	22	4575	Methylene = 0.12 H	2.65

^1^ Chemical yield was calculated based on the mass of polymer recovered after purification.

^2^ MW-R is a molecular weight calculated from the hydrodynamic radius measured by DLS. A spherical polymer is assumed, so this predominantly a useful way to compare polymers of similar structure.

^3^ The N-acetyl methyl group of the parent disaccharide was set to 3 protons. When possible, the observed integral corresponding to the example nucleophile was reported.

^4^ Triple the typical molar mass of methylamine hydrochloride was utilized.

^5^ After the second portion of chondroitin sulfate was added, these reactions were run for an additional 70 minutes (instead of 65 minutes).

#### Restoring bladder impermeability in vivo

**Fig 3:**
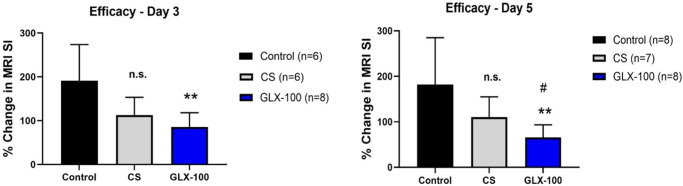
Efficacy of GLX-100 and CS in restoring impermeability to URO-MCP-1 mouse bladders treated with dilute LPS. Permeability was assessed after induction of permeability-inducing cystitis. MRI signals (post minus pre) were equated with permeability. Control vs **p<0.01, CS vs. #p<0.05.

Models of bladder permeability generally require a chemical insult to the bladder such as hydrochloric acid, acetic acid, or protamine. In this study, a well-characterized transgenic mouse model (URO-MCP-1) was used. In this model, the intravesical administration of LPS at a dose that is subnoxious to wild type mice induces the secretion of monocyte chemoattractant protein-1 (MCP-1) by the bladder epithelium. This response results in gross bladder inflammation inducing a pathology consistent with IC/BPS. It has been shown that URO-MCP-1 mice manifest symptoms of IC/BPS within 24 hrs after LPS exposure [28]. These include pelvic pain (as measured by von Frey filament stimulation), voiding dysfunction (increased urinary frequency, reduced average volume voided per micturition), and increased bladder permeability as measured by MRI and by ex-vivo TEER [29]. Fig 3 compares the MRI bladder permeability measurements of mice treated with CS or GLX-100 (Compound 5, capture agent hexylamine) at both 3- and 5-days following induction of permeability. Meaningful measurements cannot be obtained after 5 days because the bladder substantially returns to normal impermeability on day seven. At days 3 and 5 GLX-100 is effective compared to the control and at day 5 GLX-100 is more effective than CS in restoring bladder impermeability.

#### Visualizing GLX-100 on the luminal surface of the bladder

**Fig 4:**
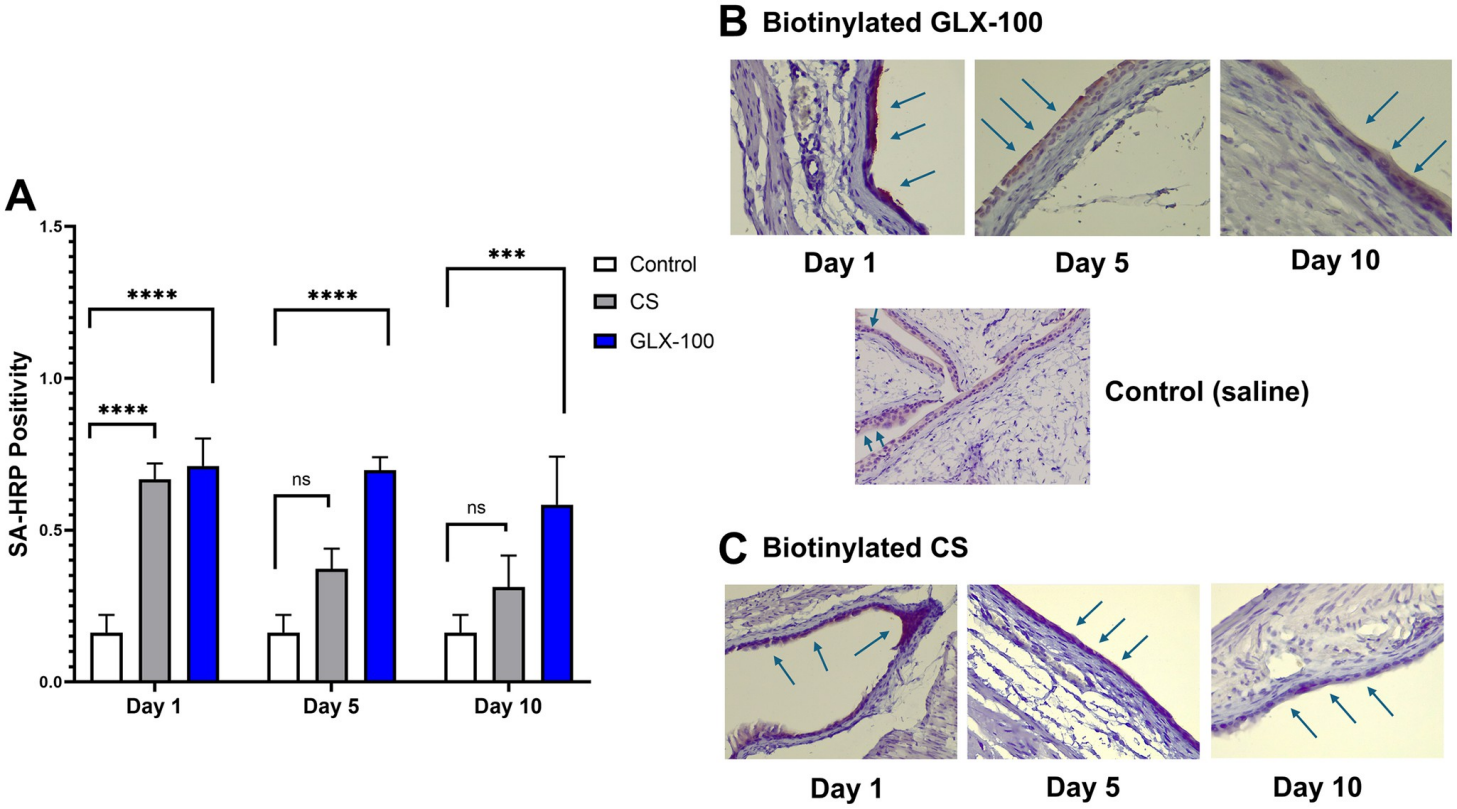
Visualizing biopolymer adherence to the luminal wall of the bladder. (A) Quantitation of biotinylated CS and biotinylated GLX-100 to the urothelium of URO-MCP1 mice (treated with dilute LPS to induce cystitis-producing permeability on day 0) on days 1, 5, and 10. Five mice were used for each of the three test groups. Controls consisted of mice that were treated with saline instead of LPS. Control vs ***p<0.001, ****p<0.0001. Bladder samples visualized via histology labeled by IHC with HRP (B) Sample images (40x) of biotinylated GLX-100 at days 1, 5 and 10. The luminal surface is oriented to the right side of all images, the signal is indicated with the arrows. (C) Sample images (40x) of biotinylated CS at days 1, 5 and 10. More details can be found in “S2 File”.

We next investigated the binding of GLX-100 to the luminal surface of the bladder by attachment of a biotin reporter. The details of the chemistry used to make these derivatives are detailed in Preparation of biotinylated materials in “S1 File”. Our controls consisted of mice treated with saline instead of LPS, they showed very little attachment of either CS or GLX-100. The samples were visualized via labelling by IHC with HRP. Normal urothelium shows no attachment perceptible to the eye. The GLX-100-treated mice showed excellent continued adherence of GLX-100 at day 10 (Fig 4). In contrast, CS was beginning to show reduced binding levels at Day 5 with even more dissolution at Day 10.

## Discussion

### Chemistry

Divinyl sulfone (DVS) is well known as a reactive crosslinker for GAGs like hyaluronic acid under alkaline conditions [30]. This chemistry has proven its clinical utility and safety in several commercial products including Synvisc-One viscosupplement for the treatment of osteoarthritis, Prevelle Silk dermal filler, and Sepragel ENT surgical dressing for ENT surgical procedures [31]. The use of DVS for the modification of soluble polymers without the formation of an extended crosslinked network has been reported but has not been extensively explored [32]. This report extends the use of DVS to the controlled oligomerization of chondroitin sulfate enabling the preparation of branched, high molecular weight sulfated GAGs that remain highly soluble and microfilterable. SuperGAGs have been prepared in a simple, 1-pot, sequential reaction procedure and functionalized with a variety of chemical motifs introduced through the capture agent. Purification by TFF using a high 100 kD MWCO membrane effectively removes all small molecule impurities as well as low molecular weight oligomers including the starting polymer. TFF also provides specific control over pH, buffer salt composition and concentration allowing exquisite control of the ionization state and salt content of the SuperGAG biopolymer product.

The characterization of SuperGAG biopolymers includes monitoring hydrodynamic radius by DLS, and molecular weight determination by SEC-MALS. ^1^H-NMR analysis of SuperGAG products has shown that the chondroitin sulfate signal remains generally unchanged but for the additional methylene resonances contributed by the divinylsulfone linking groups [24]. Complete quenching of pendant vinylsulfone groups is confirmed by the absence of vinyl resonances in the proton NMR (less than or equal to 1%.) The appearance of signals from the selected capture agent are sometimes visible and separated from that of the parent material. Purified SuperGAG can be isolated as a solid via lyophilization or maintained as an aqueous solution. Additional characterization (viscosity, FTIR, osmolality) is consistent with expectations, and methods for determining residual DVS and quenching agent have been established.

As shown in this communication (and a prior publication describing a related SuperGAG [24]) these unique biopolymers are superior to CS in producing an effective barrier capable of reducing bladder permeability as quantified by MRI. Significantly, the reduction in bladder permeability in this model persisted for the entire available observation period (5-days). Consistent with this result, histological analysis of bladder tissue isolated from mice treated with GLX-100 (modified with biotin) demonstrated a significantly longer residence time on the bladder wall than CS. The durability of enhanced barrier function may be clinically significant, since prolonged periods of impermeability may allow healing and repair of the urothelium. When compared with CS, the crosslinked higher molecular weight GLX-100 polymer restores bladder impermeability because it adheres to the bladder surface longer. The polarity of GLX-100 establishes a deep and stable layer of associated water molecules typical of naturally occurring impermeable membranes.

Recently, the scale up process has been completed at a cGMP facility and sterile clinical grade material has been produced. This material is safe and is currently deployed in a clinical trial of GLX-100 in IC/BPS patients (ACTRN12623000602628).

### Progress treating IC/BPS

IC/BPS is a complex disorder that is diagnosed by clinical criteria of longstanding pain, urinary urgency and frequency [1]. Although a number of features such as petechial bleeding on cystoscopic hydrodistension, loss of umbrella cells in the urothelium, among other features are associated with IC/BPS, none have proven to provide sufficient sensitivity and specificity to serve as definitive diagnostic criteria [33]. One feature that has been frequently reported is urothelial dysfunction [5,34] which is manifested by loss of the barrier impermeability that prevents penetration of the bladder wall by urinary solutes [5,7,10,13]. Normally the urothelium is the least permeable epithelium in the body and is mitotically quiescent [35]. Upon damage that induces increased permeability, it becomes a very rapidly dividing epithelium [36,37]. Even in the presence of virtually complete destruction, the urothelium regenerates within 14 days [38]. This property illustrates the importance of an intact urothelium to normal bladder function [39]. Several studies have clearly demonstrated that increased bladder permeability is a feature exhibited by at least a sub-set of IC/BPS patients. The fraction of patients exhibiting increased permeability will likely depend upon the stringency of the diagnostic criteria. Parsons showed significantly increased uptake of urea instilled into the bladders of IC/BPS patients [40], and Buffington showed slower excretion of fluorescein administered intravenously due to recycling from the bladder [41]. It has been shown using MRI that contrast medium leaked from the bladders of patients diagnosed with IC/BPS but not from the bladders of controls [9]. Several investigators have shown a high prevalence of a positive potassium sensitivity test (PST) in IC/BPS [42]. Gulpinar and coworkers reported that 64% of patients who met the clinical definition of IC/BPS also showed a positive potassium sensitivity test [43] as developed by Parsons. Rosenberg and Hazzard reported that of 3583 patients in a primary care setting administered the Pelvic Pain and Urgency/Frequency (PUF) questionnaire, 17.5% of women and 8.3% of men showed clinical evidence of IC/BPS, and 168/509 patients with positive PUF scores also showed a positive PST [44].

It is unclear, however, whether increased permeability is the primary cause of IC/BPS or whether it is secondary to other factors. Evidence supports that other factors prevent the normal rapid healing response of urothelium. IC/BPS shows a high comorbidity with irritable bowel syndrome (IBS) [45], and experimental studies in animals have shown that experimental induction of colon permeability with trinitrobenzene sulfonic acid (TNBS) induces bladder permeability without manipulating the bladder [26,27]. Also, induction of bladder permeability with protamine sulfate or other models reciprocally induces increased colon permeability [8]. Thus, the lower pelvic organs appear to be linked through the central nervous system in a complex manner possibly via neuronal “cross-talk” by the pelvic nerves [46,47].

Nonetheless, because the most troubling symptoms of IC/BPS of pain, urgency and frequency are common to diseases such as bladder cancer and urinary tract infections, those causes of barrier dysfunction often lead to a misdiagnosis of IC/BPS. Restoring bladder permeability is necessary to produce a meaningful therapeutic effect. So long as urinary solutes can penetrate the bladder wall and continue to induce inflammation, achieving remission is challenging. Palliative therapies to reduce pain are likely to be useful but will not lead to durable patient outcomes as monotherapies.

Restoration of bladder permeability is key to producing a durable therapeutic response. GAG replacement therapy with CS and/or hyaluronan have been used with some success, although response rates have been less than expected [48,49]. There are three potential reasons for this limited success. First, CS and HA are linear polymers. While they may coat the bladder surface, this layer is not nearly as thick and robust as that found in a normal bladder. The second reason is that the linear polymers may not remain long enough on the bladder surface to effectively maintain continuous impermeability. Third is the inherent heterogeneity of the disease such that clinical trials using a broad definition of the disease will include etiologies that may be inherently insensitive to GAG replenishment.

## Conclusion

The data presented in this report support the assertion that GLX-100 will be a more effective agent for GAG replenishment therapy than CS for treating IC/BPS patients with a bladder permeability etiology. Efficacy of GLX-100 was established in a leading mouse model of bladder permeability, and the safety of GLX-100 has been established in a set of standard biocompatibility studies. GLX-100 has been prepared reproducibly on a large scale in a GMP setting and is presently being evaluated in a clinical trial enrolling IC/BPS patients. GAG replenishment therapy with GLX-100 may provide a new therapeutic option for patients suffering with IC/BPS.

## Supporting information

S1 File The bulk of supporting information. Includes materials, equipment, protocol for dynamic light scattering experiments, S1 Table 1: Summary of safety testing, limitations of the general method, preparation of biotinylated polymers, S1 Table 2: Raw data for Fig 1, extended characterization for GLX-100, S1 Figure 1: ^1^H NMR spectra of GLX-100, S1 Figure 2: Images of gadolinium contrast MRI experiments.
(PDF)

S2 File Data from histology experiments. Binding affinity: Histology streptavidin-horseradish peroxidase (SA-HRP) data. S2 Table 1: Horseradish peroxidase (HRP) affinity (positivity) values regarding binding of SA-HRP to biotinylated GLX-100 or biotinylated chondroitin sulfate (CS) that were administered to URO-MCP-1 mouse bladders via an intravesical catheter. Control mouse bladders were only administered saline. Histological slide examples of saline-treated control, biotinylated-CS-treated, or biotinylated-GLX-100-treated URO-MCP-1 mouse bladder urothelium’s stained with SA-HRP at either 40x or 10x magnifications. Biotinylated-CS- or biotinylated-GLX-100-treated mouse bladders were obtained on days 1, 5 and 10 post-LPS exposure. Control saline bladders were obtained on day 1 post-LPS exposure.
(PDF)
