# Supplementary material for: Development of a unique crosslinked glycosaminoglycan for soft tissue repair: Treatment of interstitial cystitis/bladder pain syndrome
Source: PLoS One. 2025 Jan 24;20(1):e0317790. doi: 10.1371/journal.pone.0317790 (PMC11760559; doi:10.1371/journal.pone.0317790)
Supplement: S1 File — Includes materials, equipment, protocol for dynamic light scattering experiments, S1 Table 1: Summary of safety testing, limitations of the general method, preparation of biotinylated polymers, S1 Table 2: Raw data for Fig 1, extended characterization for GLX-100, S1 Figure 1: 1H NMR spectra of GLX-100, S1 Figure 2: Images of gadolinium contrast MRI experiments. (PDF) [file pone.0317790.s001.pdf]

Table of Contents

Materials	1
Equipment	2
Protocol for dynamic light scattering	3
Safety testing	4
Limitations of the general method	5
Preparation of biotinylated polymers	5
Raw data for figure 1	6
More characterization for GLX-100	7
Images of gadolinium contrast MRI experiments	8

Materials

Chondroitin sulfate was injection grade (F0511, M_w = ~12 kD, bovine source) purchased from Bioiberica. Water was cell-culture grade, endotoxin free (Cytiva, SH30529.FS). Sodium chloride was from Spectrum Chemical MFG Corp S1248. Divinylsulfone was from Millipore Sigma V3700. 1N aqueous sodium chloride SS266-1 and 1N aqueous hydrochloric acid SA47-500 were from Fisher Chemical. 2-Propanol was from Fisher Chemical A451-4. pH paper was from Hydrion. Dulbecco's phosphate-buffered saline was from Gibco 14190136. Lipopolysaccharide was from Millipore Sigma E. Coli 055: B5. Mouse on Mouse ImmPRESS® Polymer kit, Peroxidase was from Vector Laboratories MP-2400. Antigen Unmasking Solution, Citrate-based, was from Vector Laboratories H-3300. Peroxidase blocking reagent Bloxall® was from Vector Laboratories SP-6000. Streptavidin, Peroxidase, R.T.U. was from Vector Laboratories SA-5704. Vector® NovaRed® Substrate kit, Peroxidase was from Vector Laboratories SK-4800. Hematoxylin QS Nuclear Counterstain was from Vector Laboratories H-3404.

Capture Agents: 2-(2-methoxyethoxy)ethanamine was from Millipore Sigma 901158. Methylamine hydrocholoride was from Sigma Aldrich M0505. n-Amylamine was from Thermo Scientific Chemicals AC173940050. 1-Butylamine was from Thermo Scientific Chemicals AAL03575AE. Hexylamine was from Acros Organics 204721000. n-Heptylamine was from Thermo Scientific Chemicals AC155460250. n-Methylhexylamine was from Matrix Scientific 007627. sec-Butylamine was from TCI B0708. 3-Dimethylaminopropylamine was from Acros Organics AC115900010. 2-Ethoxyethylamine was from TCI E0223. Benzylamine HCl was from Millipore Sigma B5136. Pyrrolidine was from Alfa Aesar A14852AE. 4-Methylpiperidine 99% was from Acros Organics AC127511000. 4-Methylpiperidine-4-ol hydrochloride was from Chem Scene CS-B1024. Cyclohexylamine was from Thermo Scientific Chemicals AAA15851AE. Hexamethyleneimine was

from Thermo Scientific Chemicals AC120590050. Glycine hydrochloride was from Sigma Life Science G2879. β-Alanine was from Thermo Scientific Chemicals AAA1666530. L-Serine was from Sigma Life Science S4311. L-Phenylalanine was from Thermo Scientific Chemicals AAA1323814. L(+)-Lysine was from Acros Organics AC303341000. L(+)-Arginine was from Acros Organics AC104991000. 3-Aminopropanoic acid (beta-alanine, 98%) was from Thermo Scientific Chemicals AAA1666530. Glycyl-DL-phenylalanine was from TCI G0135. L-Cysteine was from Thermo Scientific Chemicals AAA1043518. N-Acetyl-L-cysteine, 98% was from Thermo Scientific Chemicals AC160280250. DL-Homocysteine was from TCI H0159. Glutathione reduced form was from TCI G0074. Tryptamine hydrochloride was from Acros Organics AC157980050. 4-(Aminomethyl)benzoic acid was from Thermo Scientific Chemicals AAB2351914. Hydroxylamine hydrochloride was from Thermo Scientific Chemicals AC270101000. (2-Hydroxyethyl)hydrazine was from Thermo Scientific Chemicals AA4379309. Acethydrazide was from Thermo Scientific Chemicals AC102350250. Valeric acid hydrazide, 95% was from Thermo Scientific Chemicals AAL0155506. N-(2-Aminoethyl)biotinamide was from Combi Blocks QB-7649. Biotin-peg7-amine was from Combi-Blocks QI-1914. Propargyl-PeG3-amine was from Ambeed A755212. 11-Azido-3,6,9-trioxaundecan-1-amine was from TCI A2363. (R)-3-Amino-1, 2-propanediol was from Thermo Scientific Chemicals AC397880050. 1,6-Hexamethydiamine was from Fisher Science Education S25350. Spermidine, 99% was from Thermo Scientific Chemicals AAA1909603. 1-Hexanethiol, 96% was from Thermo Scientific Chemicals AC215270050. 2-Ethylhexyl thioglycolate was from TCI T0612. 2-Mercaptoethanol was from Thermo Scientific Chemicals AAA1589030. 2,2,2-Trifluoroethylamine was from TCI T1169. (+/-)-2-Aminoheptane, 98% was from Thermo Scientific Chemicals AAB2286314. 4-Aminothiomorpholine 1,1-Dioxide was from TCI A1798. Dihexylamine, 99% was from Acros Organics AC407591000. N-Octylamine in 1 ml water, HCl to neutral pH was from Thermo Scientific Chemicals AC129490050. L-Methionine, 98% was from Thermo Scientific Chemicals AC166160250. 4-Aminobenzoic Acid was from TCI A0269. Imidazole, 99% was from Thermo Scientific Chemicals AC122021000. Indole, 99% was from Thermo Scientific Chemicals AC122150250. Pyrazole, 98%, pure was from Thermo Scientific Chemicals AC131740250. 1-Aminopiperidine was from TCI A2241. 2-(Aminooxy)ethanol was from Chem Scene CS-W011361. 4-Aminomorpholine was from TCI A0832. O-Phosphorylethanolamine, 98% was from Thermo Scientific Chemicals AC367420050. Taurine, 99% was from Thermo Scientific Chemicals AAA1240322. Tris(hydroxymethyl)aminomethane was from Fisher Chemical T395-100. Polyethyleneimine, 2.5k was from Polysciences, Inc. 24313-2.

Equipment

Materials were weighed out on a Mettler Toledo ML204T analytical balance. Liquids were aliquoted using Eppendorf® Research plus™ adjustable volume pipettors. Microfiltrations were performed with Cytiva Whatman™ Uniflo syringe filters (09-928-062). Polymers were purified on a Spectrum® KR2i/KMPi Tangential Flow Filtration system using either 100 kilodalton Repligen D02-E100-05-N or 3 kilodalton Repligen D02-E003-05-N polyethersulfone cartridges. Lyophilization was done with a Labcono FreeZone -105 ^{o}C 4.5 liter Cascade benchtop freeze dryer system. Dynamic light scattering was measured in a quartz cuvette (Wyatt Technology WNQC01-

00) on a Wyatt Labs DynaPro Nanostar running Dynamics Software version 7.10.1.21. Quartz Cuvette was from Wyatt Technology WNQC01-00, JC-834. Nuclear Magnetic Resonance imaging was gathered on a 400 MHz JOEL magnet with a Royal probe ECZ using Delta software; a relaxation time of 5 seconds was utilized. Molecular weight determination was performed using two Agilent PL aquagel-OH Mixed-H columns, 8 μm particle size columns preceded by a PL aquagel-OH guard, 0.01 molar sodium nitrate and 0.01 molar sodium phosphate monobasic in water adjusted to pH 7.0 with sodium hydroxide mobile phase, a Wyatt Technologies HELEOS II multi-angle light scattering detector and a Wyatt Technologies T-rEX refractive index detector using a literature dn/dc value of 0.1427 mL/g. MRI experiments were conducted on a 7 Tesla 30 cm-bore Bruker Biospec system from Bruker Biospin Corporation. MRI contrast agent Gd-DTPA was Magnevist from Bayer Inc. Regions of Interest in the MRI data were displayed on Paravision, V 5.0 from Bruker Biospin. HistoBond® Plus slides were from Statlab Medical Products. The steamer was from AROMA. Quantification of histology images was performed on Aperio ImageScope from Leica Biosystems.

Protocol for dynamic light scattering

Fill the quartz cuvette with isopropanol and let it sit for 5 minutes. After 5 minutes, transfer the isopropanol into the proper waste container and allow the cuvette to dry fully.

Turn on the DynaPro Nanostar DLS by flipping the power switch to the on position. Wait for the small screen to display a graph indicating that the instrument has loaded its firmware. Click the Dynamics icon on the computer screen, open a new file, and save it with the name of the sample to be analyzed. Establish a connection between the instrument and computer by pressing the "plug" icon; a small circle icon in the top left-hand corner of the screen will turn green when the connection is successful, and the connection icon will show as plugged in.

Weigh out between 4–10 mg of the test material into a 15 ml centrifuge tube, label each tube with the sample name, and record the sample mass. Add the appropriate amount of DPBS to the 15 ml tube to create a 2 mg/ml solution (0.2% w/w). Mix each sample with a vortex genie until it is homogenous, then transfer the solution into a 3 ml syringe and place a 0.2-micron syringe filter on the syringe.

To ensure the cuvette is clean, add 3 drops of DPBS to the cuvette, tap the cuvette gently to remove bubbles, and use an air duster to clean any dust particles from the outside of the cuvette. Place the cuvette into the DLS with the letters facing the user and allow the cuvette to come up to temperature for ~30 seconds. Start data collection by pressing the green circle icon in the top left-hand corner. Verify the results after data collection.

Remove the cuvette, remove the DPBS solution and use a 100-microliter pipette to remove residual solution. Load the polymer test samples by pushing three drops from the syringe through the 0.2-micron syringe filter into the cuvette. Tap the cuvette to remove any bubbles, clean the

outside with an air duster, and place the cuvette back into the DLS. After waiting ~30 seconds, start data collection. Rename the sample with the correct name by right-clicking on the sample name and typing the sample name. Go to the table tab and change the 'Solvent' to PBS using the drop-down menu. Repeat this process for each test sample twice to generate an N=2 for each compound. Record the Radius, R-MW, and S-MW. Clean the cuvette by running it under DIW and verify cleanliness by running a PBS sample before testing another sample.

S1 Table 1. Summary of safety testing.

Test	Method (ISO number)	Result
MTT Cytotoxicity	10993-5	No cytotoxic potential
Acute Systemic Toxicity	10993-11	No mortality or evidence of systemic toxicity
Maximization Sensitization	10993-10	No evidence of delayed dermal contact sensitization
Intramuscular Implantation	10993-6	Non-reactive
Urinary Bladder Irritation	10993-23	Nonirritant to the urinary bladder
Reverse Mutation	10993-3	Non-mutagenic

Evaluation of GLX-100 was performed at NAMSA, Northwood, Ohio or Labcorp, Bedford, Massachusetts. These are both ISO-accredited facilities.

Limitations of the general method

The polymer preparation described in this paper does have limitations. Not all capture agents resulted in polymeric products with the absence of vinylsulfone signals in the ^{1}H NMR. A review of the molecules that weren't successful suggests that those failures are the results of the following agent properties in this order: solubility in the reaction solvent, molecular weight, and nucleophilicity.

Capture agents that were not successful at quenching the vinyl signals from the ^{1}H NMR using the provided protocol include the following: 2,2,2-trifluoroethylamine, 2-aminoheptane, 4-aminothiomorpholine-1,1-dioxide, dihexylamine, N-octylamine, methionine, 4-aminobenzoic acid, imidazole, indole, pyrazole, 1-aminopiperidine, 2-aminooxyethanol, 4-aminomorpholine, O-phosphorylethanolamine, taurine, tri(hydromethyl)aminoethane, polyethyleneimine 2.5k.

Preparation of biotinylated polymers

Note: This protocol uses a variant synthesis that adds two more stages to the polymer synthesis. Referencing figure 1, after stage 2 the reaction is diluted then another portion of DVS is added. After a pause the capture agent is added. The net result of this is differences in final molecular weight, and a larger proportion of capture agent located on the surface. This was disclosed in patent WO20211188882.

A mixture of chondroitin sulfate (2.86 g), of Biotin-PEG7-amine (0.36g, CAS 1334172-76-7), 4-(4,6-dimethoxy-1,3,5-triazin-2-yl)-4-methylmorpholinium chloride (0.168g, DMTMM) and of DPBS (24.8 mL) was vortexed until homogeneous and magnetically stirred at ambient temperature overnight. The reaction was diluted with DPBS to ~120 mL total volume. This solution was subjected to 0.45 micron filtration and purified via tangential flow filtration (3 kD filter). The retentate was exchanged against 5 volumes of DPBS then 5 volumes of water. The retentate solution was again filtered (0.2 micron) and lyophilized to provide 735 mg (75%) of a white solid. ^{1}H NMR (D_2O, 400 MHz) δ 4.7 – 3.3 (m, 12H), 3.03 (m, 0.023H), 2.8 (m, 0.028H), 2.3 (t, 0.17 H), 1.9 – 5.5 (m, 0.19 H), 1.5 – 1.4 (m, 0.077 H); DLS hydrodynamic radius = 4.2 nM, MW-R = 28 kD.

A solution of sodium chloride (47.1 mg) water (4.83 g) and biotinylated chondroitin sulfate (158 mg, described above) was prepared. Once everything was dissolved, divinylsulfone (70 mg) was added followed by aqueous sodium hydroxide (0.537 mL, 1N NaOH). The resulting yellow solution was stirred magnetically. After 15 minutes another portion of biotinylated chondroitin sulfate (473 mg, described above) was added. The resulting suspension was mixed using a Vortex Genie until the polymer was completely dissolved. After 105 minutes 4 mL water was added to the reaction. After another 15 minutes divinylsulfone was added (57.8 μL), another 15 minutes of stirring, then hexylamine (110 μL) was added and the reaction stirred for an additional 15 minutes. The pH of the solution was adjusted to 7 with 1N aqueous hydrochloric acid and diluted to a volume of ~40 mL with DPBS. This solution was subjected to 0.2 micron filtration and purified via tangential flow filtration (100 kD filter). The retentate was exchanged against 8 volumes of

DPBS then 5 volumes of water. The retentate solution was again filtered (0.2 micron) and lyophilized to provide 375 mg (59%) of a white solid. ^{1}H NMR (D_2O, 400 MHz) δ 4.7 – 3.3 (m, ~12H), 3.03 (m, 0.023H), 2.82 (m, 0.030H), 2.3 (t, 0.20 H), 2.08 (two overlapping broad singlets, 3H), 1.8 – 1.6 (m, 0.51 H), 1.5 – 1.3 (m, 1.1 H), 0.9 (br t, 0.52 H); DLS hydrodynamic radius = 19 nM, MW-R = 420 kD. ^{1}H NMR spectroscopy confirmed that there was the same degree of biotin conjugation after crosslinking.

S1 Table 2. Raw data for Figure 1, Reaction times, chemical yield and DLS data.

Time (min)	Yield (g)	% Yield	Average Hydrodynamic Radius	Average MW-R
**55**	0.7400	54%	9.55	666.8
**65**	0.8362	61%	12.85	1332.65
**75**	0.7871	57%	15.3	1998.3
**85**	0.7232	52%	15.75	2138.85
**95**	0.7803	57%	23.45	5424.20
**105**	0.7894	57%	29.7	9372.75

Extended characterization data for GLX-100 (a hexyl derivative)

Following the general procedure in the paper utilizing hexylamine (202 μL, 1.54 μmol) of as the capture agent provides 780 mg (57%) of a white solid: ^{1}H NMR (D_2O, 400 MHz) δ 4.7 – 3.3 (m, 12H), 3.17 (m, 0.11 H), 2.08 (two overlapping broad singlets, 3H), 1.75 (br m, 0.14 H), 1.5 – 1.3 (br m, 0.36 H), 0.89 (m, 0.22 H); IR (ATR) 3280 (OH, br), 2950 (C-H), 1600 (C=O), 1400, 1380, 1050 (S=O); SEC-MALS M_n = 179 kD, M_w = 965 kD, PDI = 5.4; DLS hydrodynamic radius = 23.5 nm, MW-R = 5420 kD.

S1 Figure 1. ^{1}H NMR spectra for GLX-100

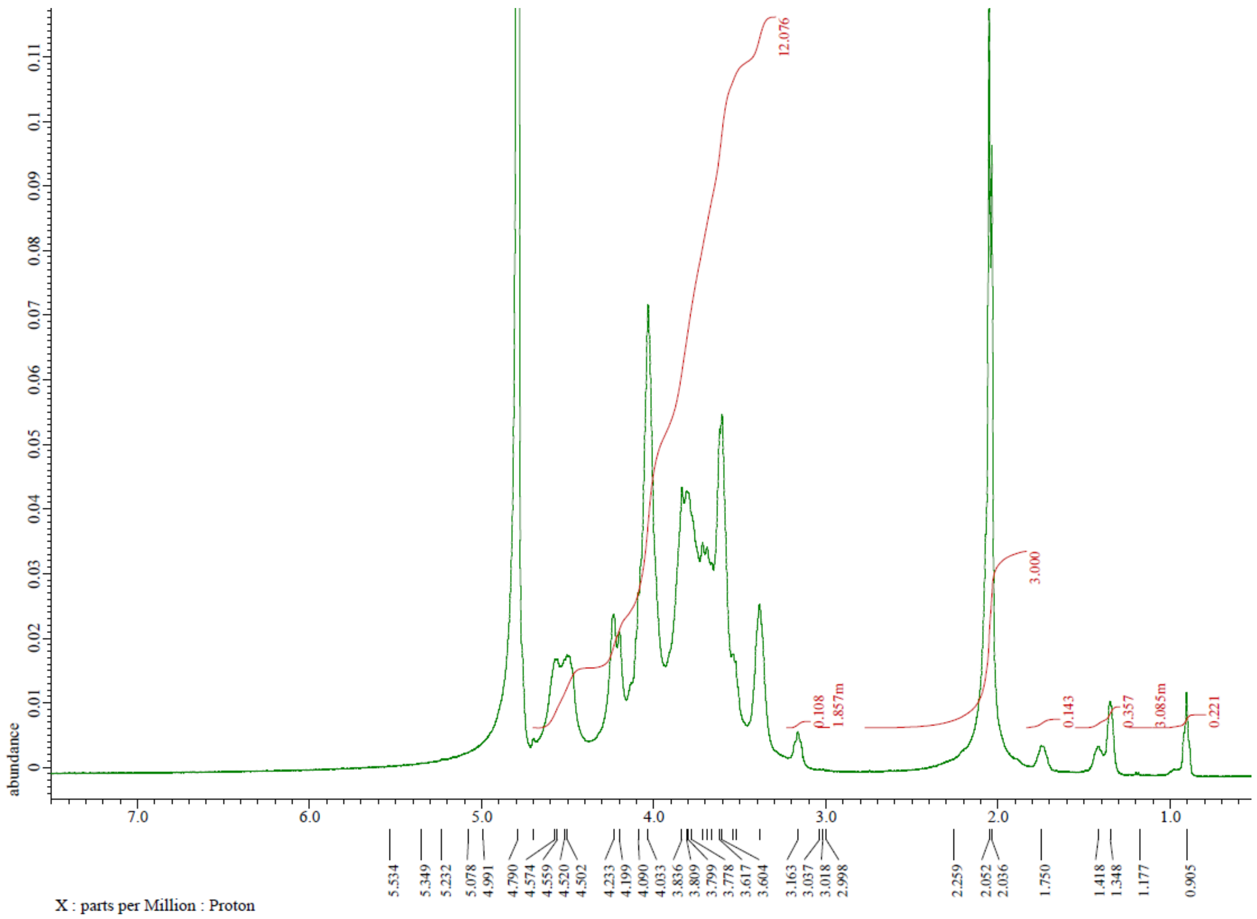

Solvent: D_2O; the methyl group of the N-Ac methyl group was set to 3 protons.

S1 Figure 2. Images of gadolinium contrast MRI experiments.

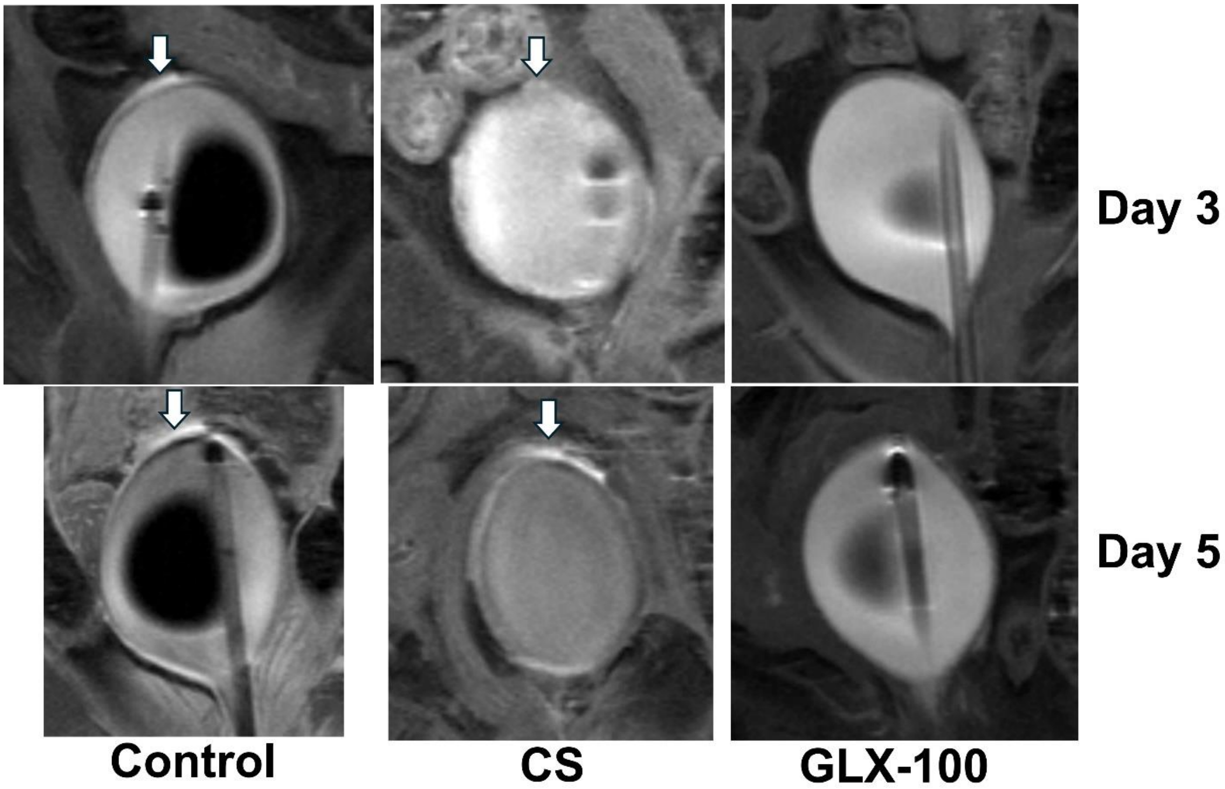

Magnetic resonance images of URO-MCP-1 mouse bladders subjected to LPS (alone – Control), LPS and chondroitin sulfate (CS), or LPS and GLX-100 on days 3 or 5 post-LPS exposure. White arrows indicate a “bright” MRI signal intensity due to leakage of MRI contrast agent, Gd-DTPA, through the bladder urothelium.
