# Supplementary material for: Development of a unique crosslinked glycosaminoglycan for soft tissue repair: Treatment of interstitial cystitis/bladder pain syndrome
Source: PLoS One. 2025 Jan 24;20(1):e0317790. doi: 10.1371/journal.pone.0317790 (PMC11760559; doi:10.1371/journal.pone.0317790)
Supplement: S2 File — Binding affinity: Histology streptavidin-horseradish peroxidase (SA-HRP) data. S2 Table 1: Horseradish peroxidase (HRP) affinity (positivity) values regarding binding of SA-HRP to biotinylated GLX-100 or biotinylated chondroitin sulfate (CS) that were administered to URO-MCP-1 mouse bladders via an intravesical catheter. Control mouse bladders were only administered saline. Histological slide examples of saline-treated control, biotinylated-CS-treated, or biotinylated-GLX-100-treated URO-MCP-1 mouse bladder urothelium’s stained with SA-HRP at either 40x or 10x magnifications. Biotinylated-CS- or biotinylated-GLX-100-treated mouse bladders were obtained on days 1, 5 and 10 post-LPS exposure. Control saline bladders were obtained on day 1 post-LPS exposure. (PDF) [file pone.0317790.s002.pdf]

Binding Affinity – Histology Streptavadin-Horseradish Peroxidase (SA-HRP) Data

Biotinylated- GLX-100

Biotinylated-CS

Control (Saline)

S2 Table 1: Horseradish perodoxidase affinity (positivity) values

**Control (Saline)**	**Sample 1**	**2**	**3**	**4**	**5**
**Day 1**	0.163	0.092	0.191	0.124	0.242
**Biotinylated CS**	Sample 1	2	3	4	5
**Day 1**	0.592	0.663	0.671	0.732	0.691
**Day 5**	0.323	0.304	0.382	0.391	0.473
**Day 10**	0.152	0.361	0.373	0.412	0.273
**Biotinylated GLX-100**	Sample 1	2	3	4	5
**Day 1**	0.613	0.831	0.679	0.652	0.779
**Day 2**	0.641	0.716	0.682	0.691	0.756
**Day 3**	0.800	0.648	0.500	0.379	0.591

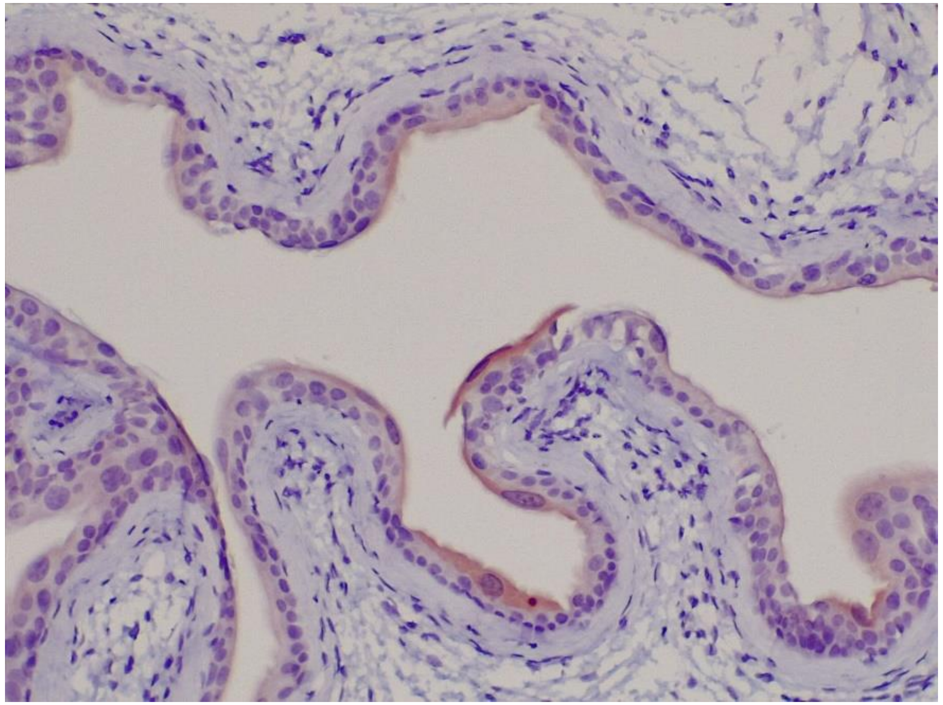

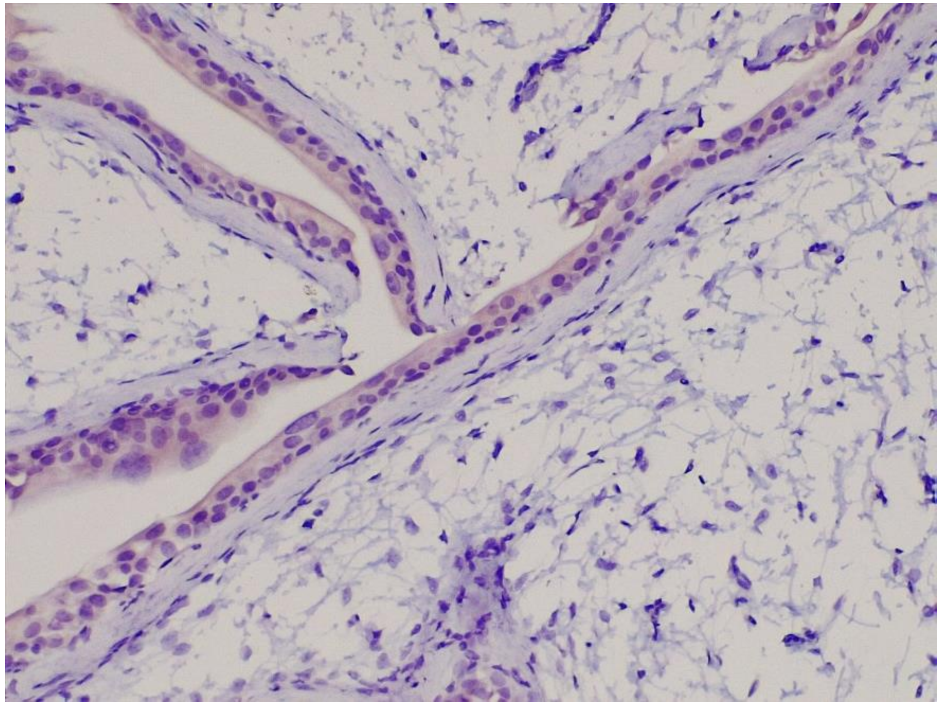

Baseline (saline) controls
(40x magnification)

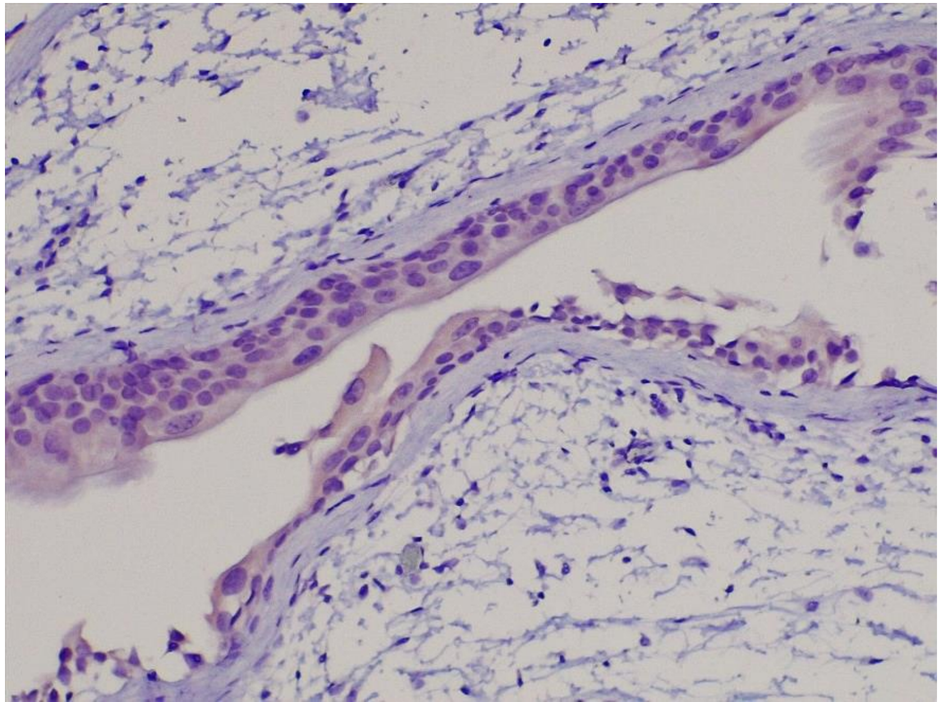

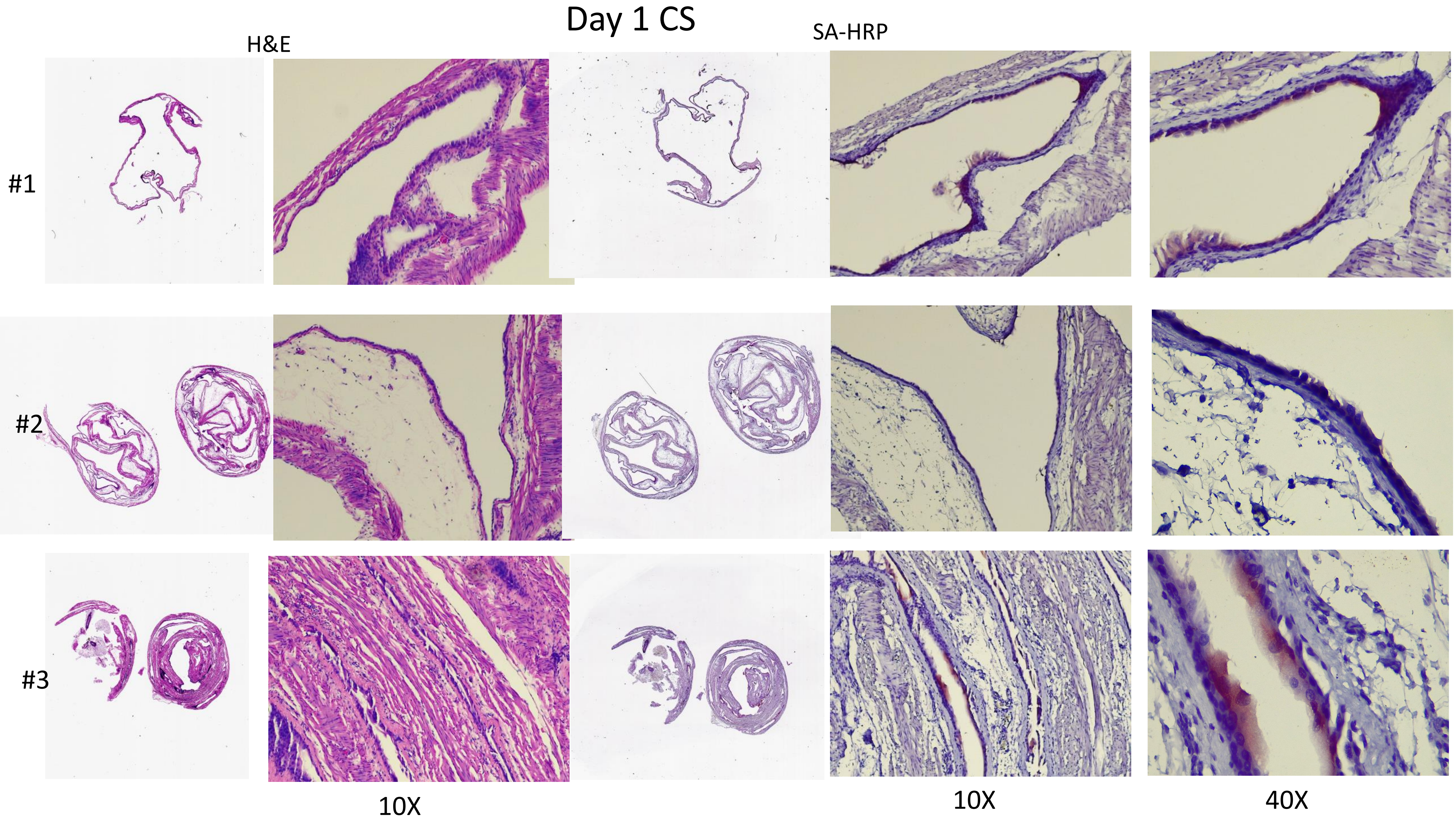
Day 1 CS
H&E
SA-HRP
#1
#2
#3
10X
10X
40X

Day 1 #1

CS

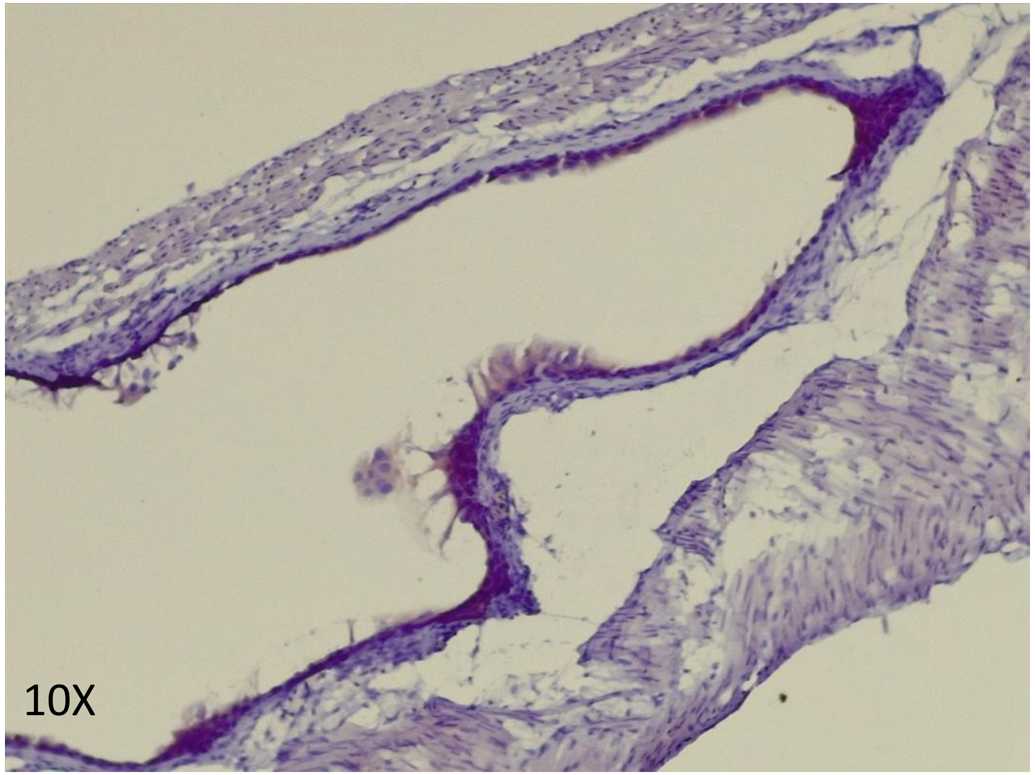

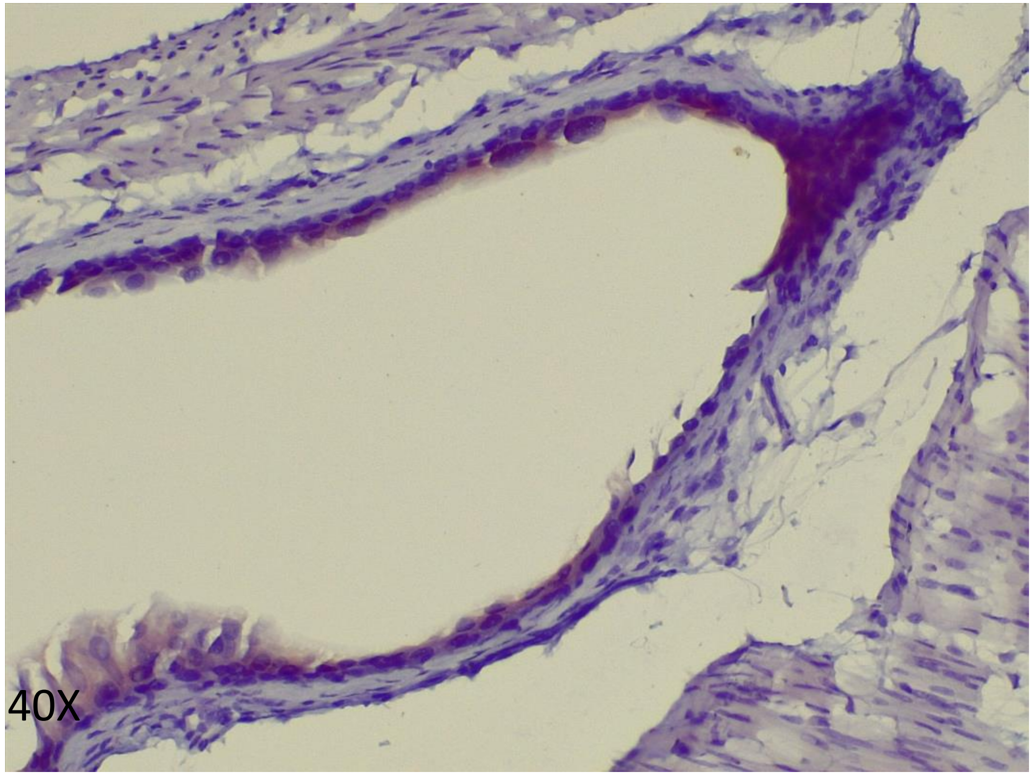

SA-HRP

Day 1 #2
CS

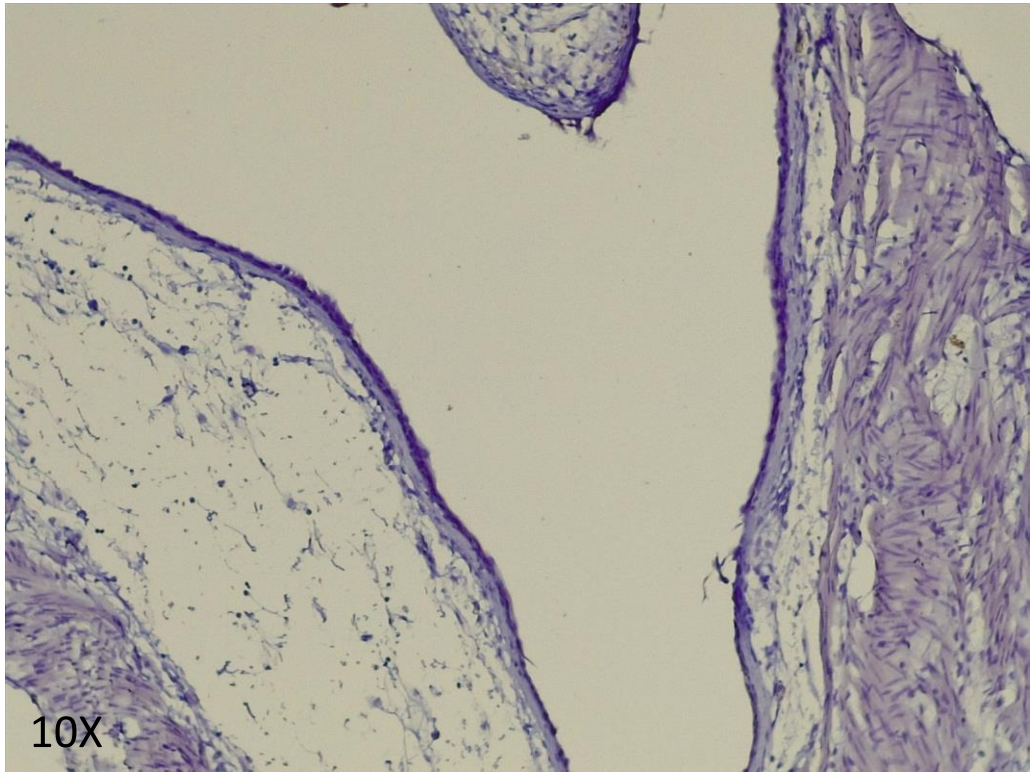

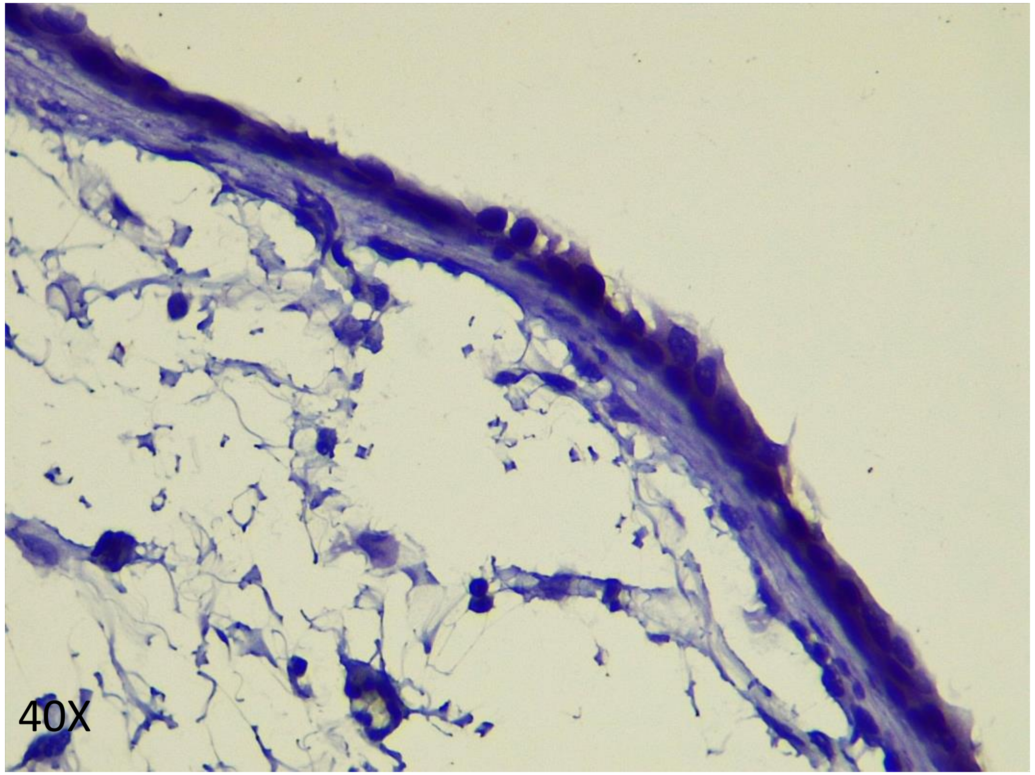

SA-HRP

Day 1 #3

CS

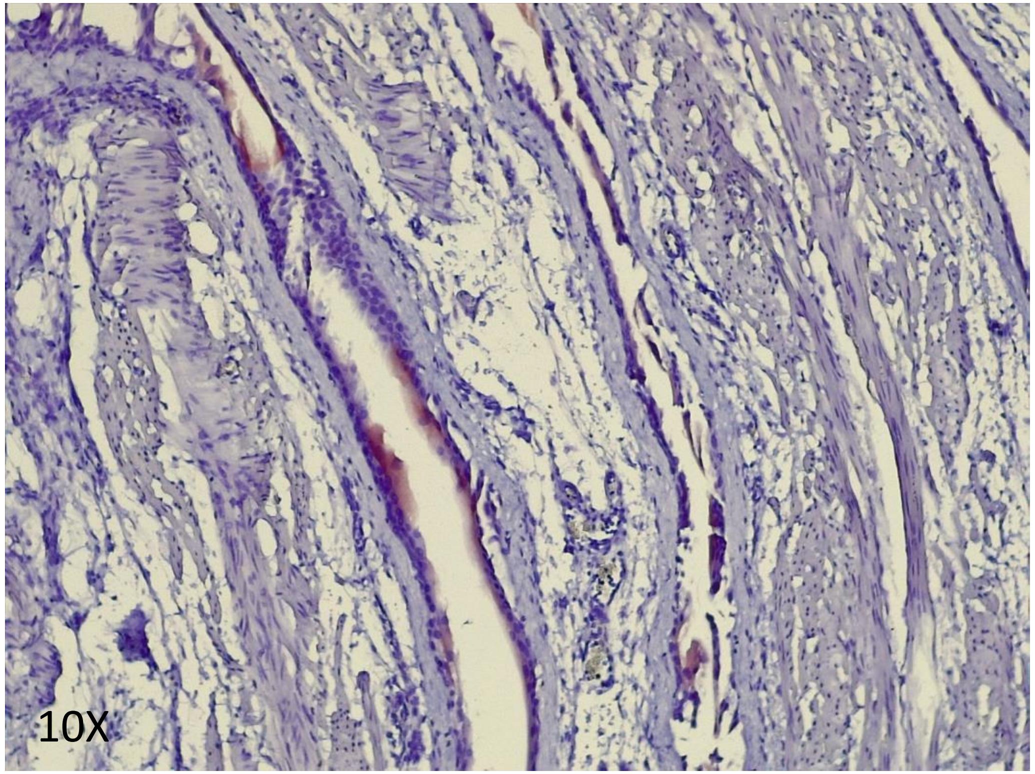

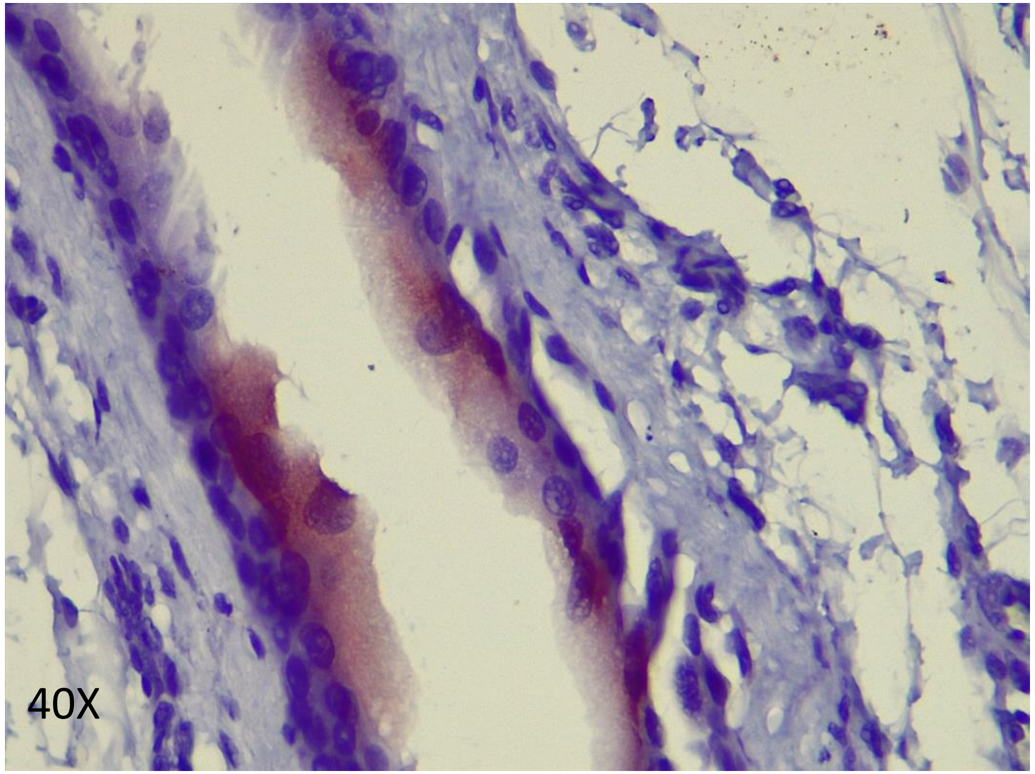

SA-HRP

Day 5 CS

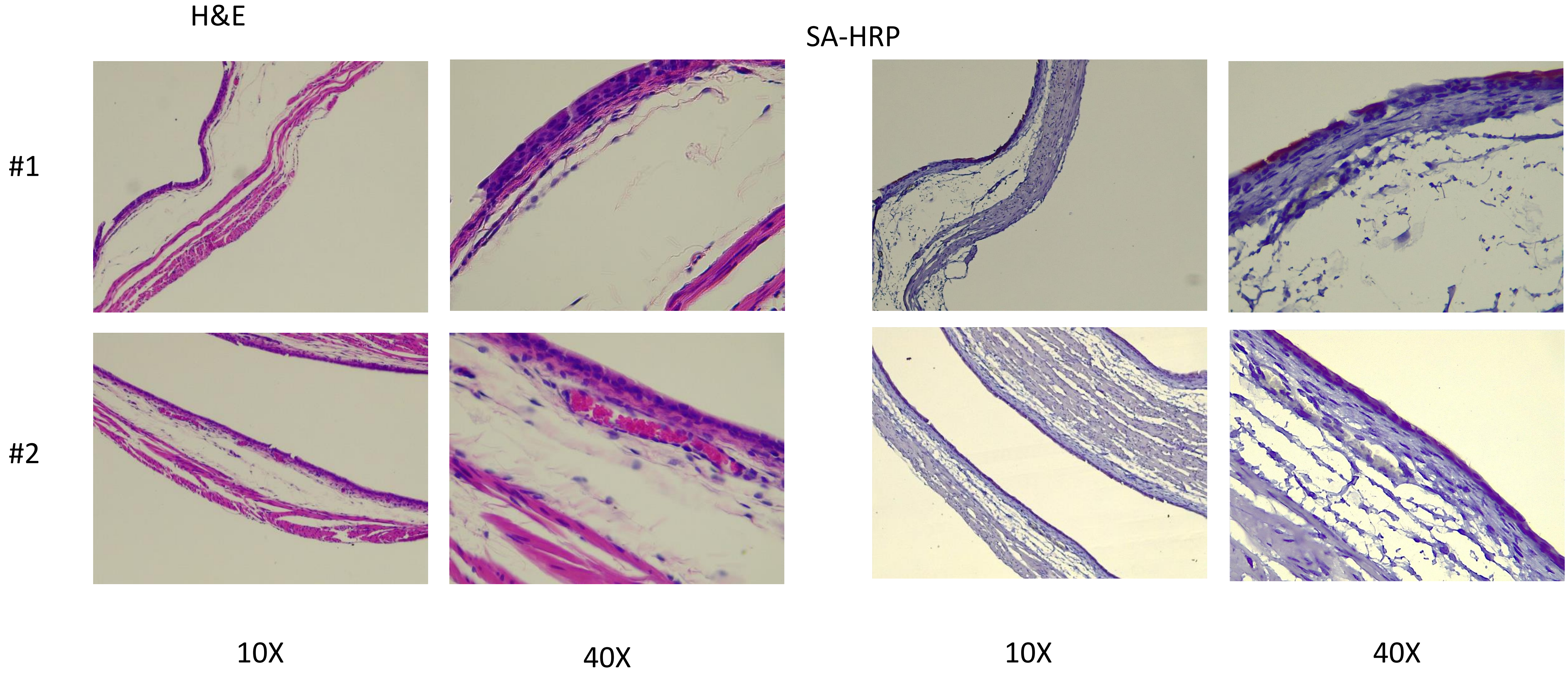

Day 5 #1
CS

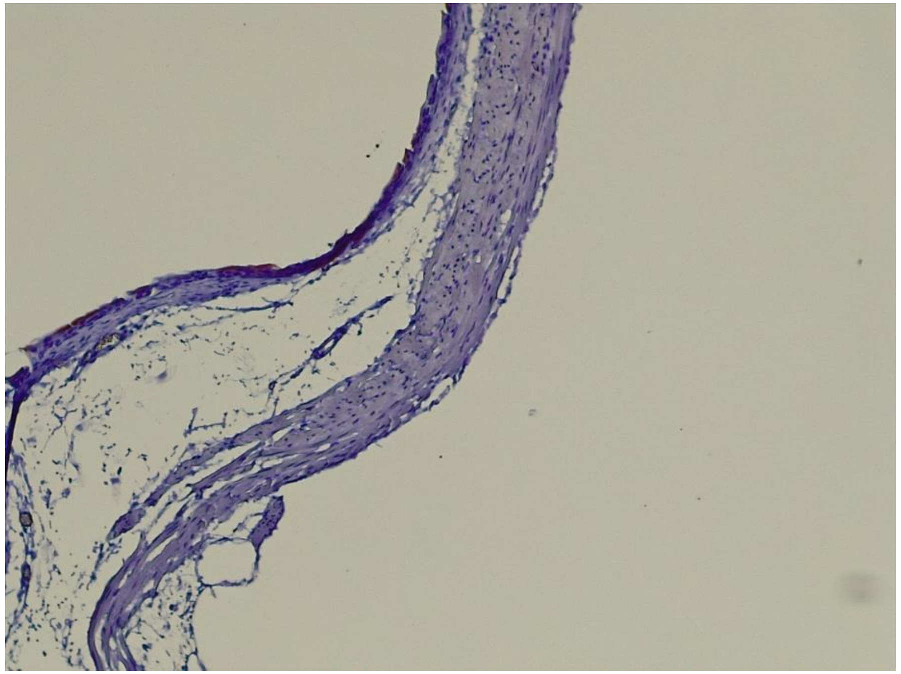

10X

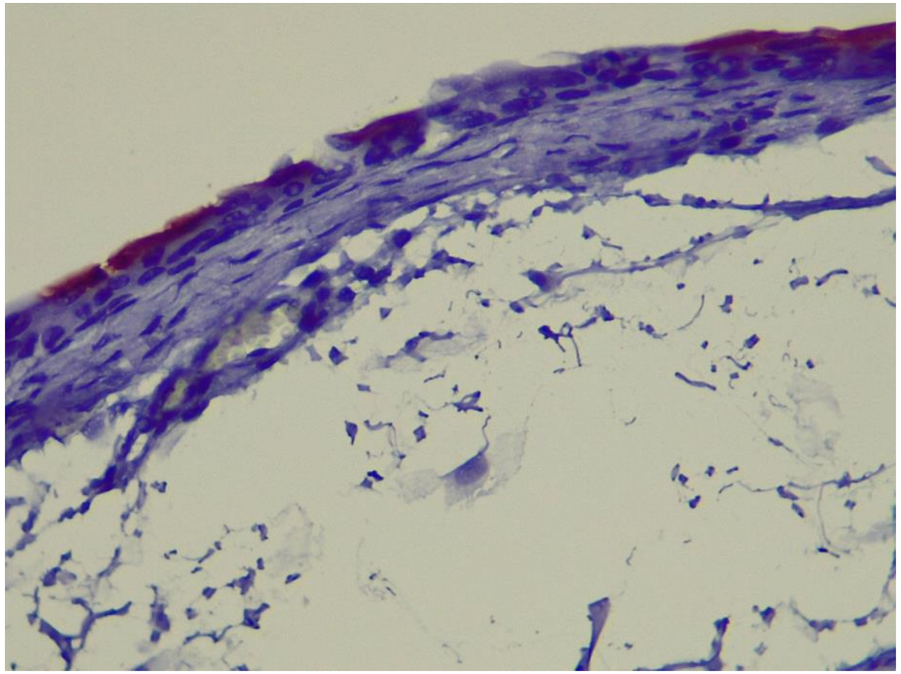

40X

SA-HRP

Day 5 #2
CS

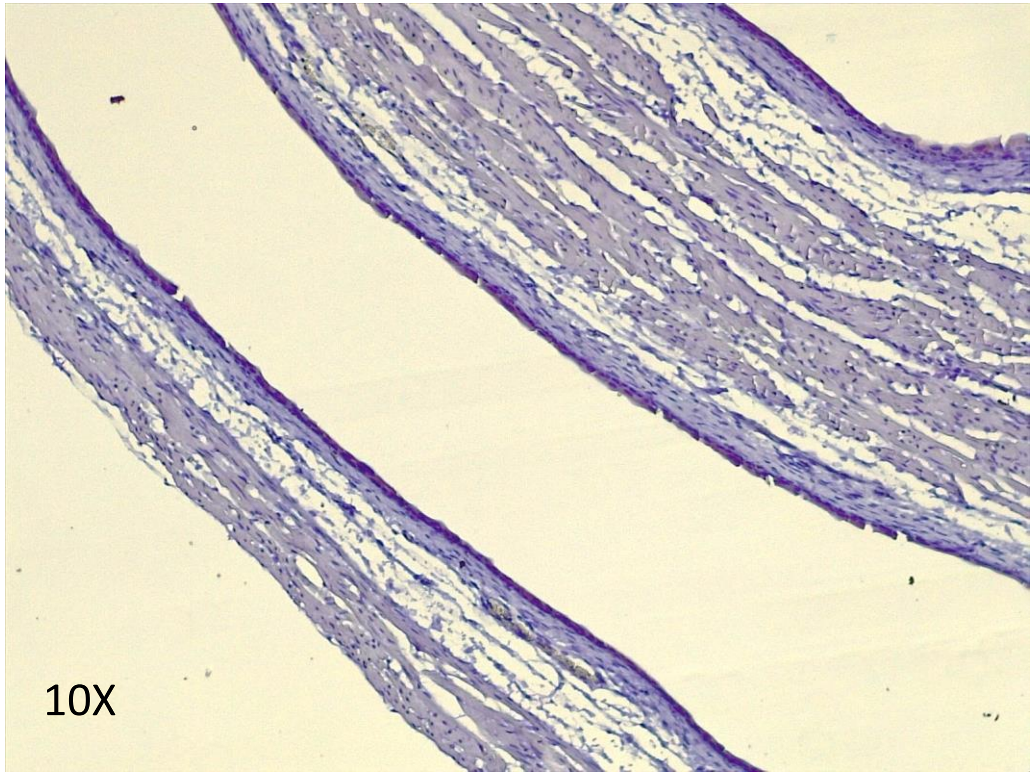

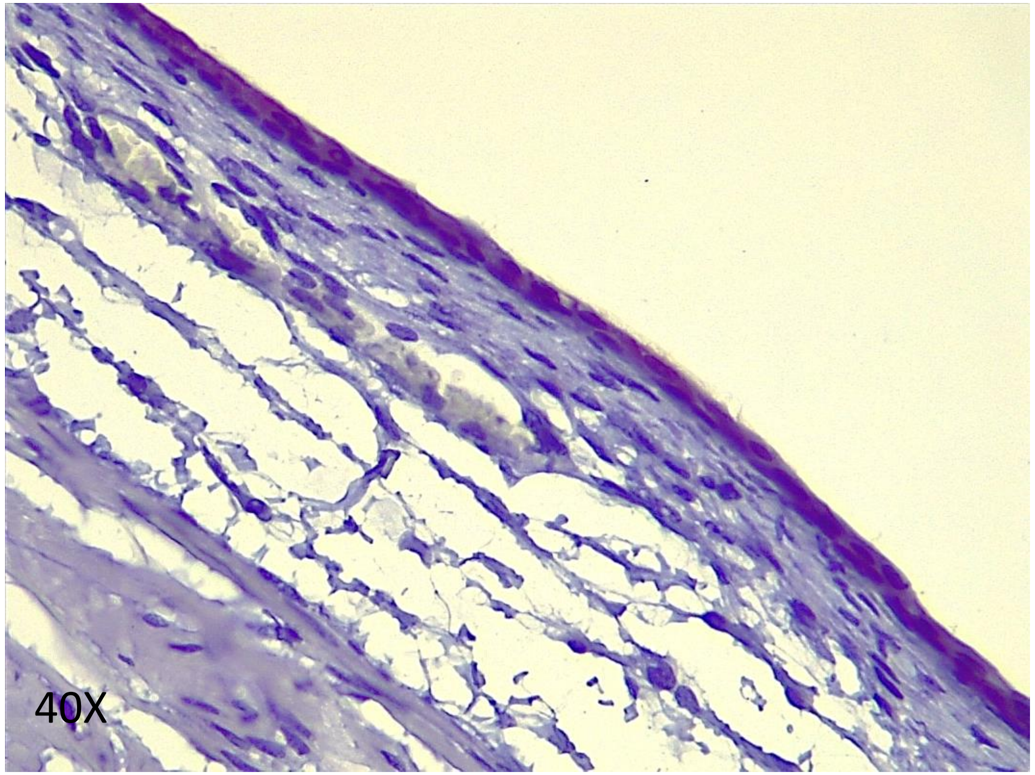

SA-HRP

Day 10 CS

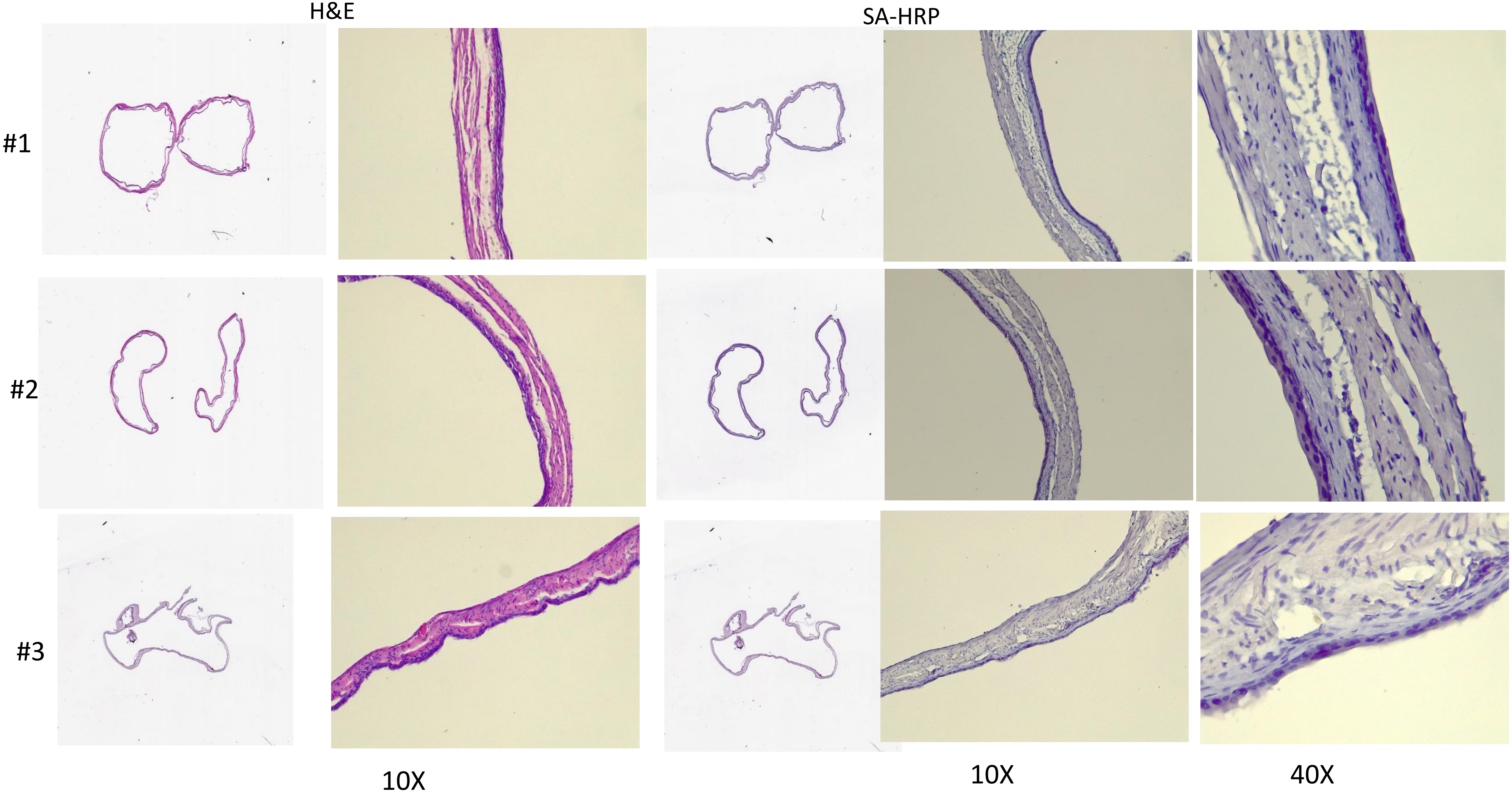

Day 10 #1

CS

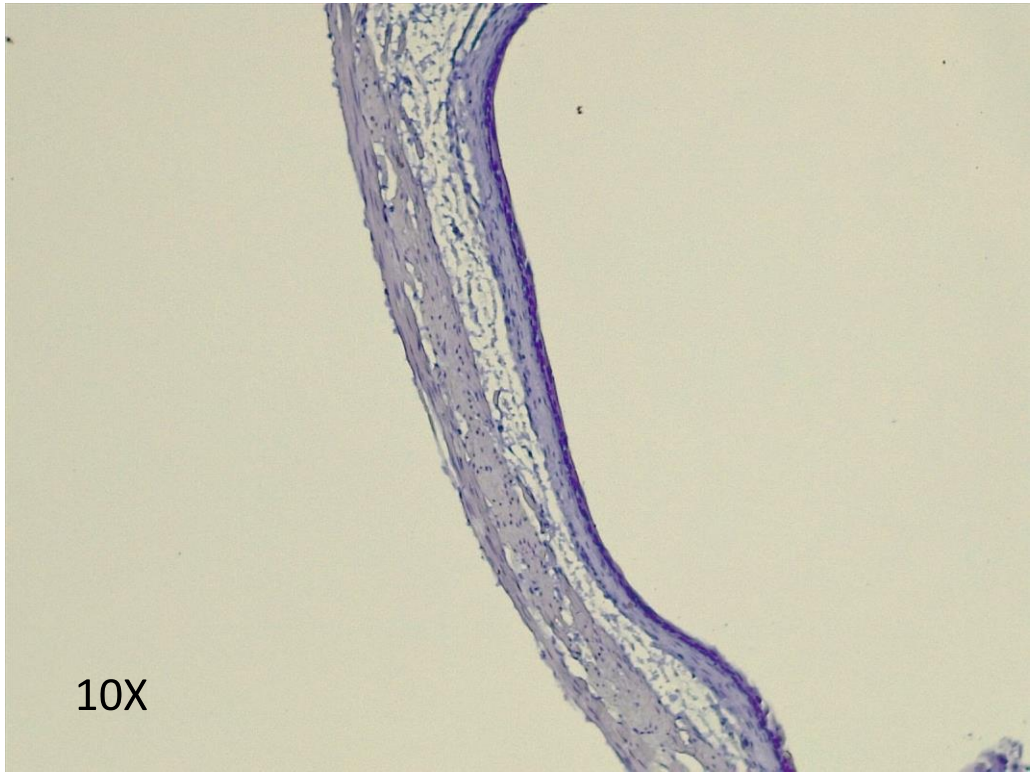

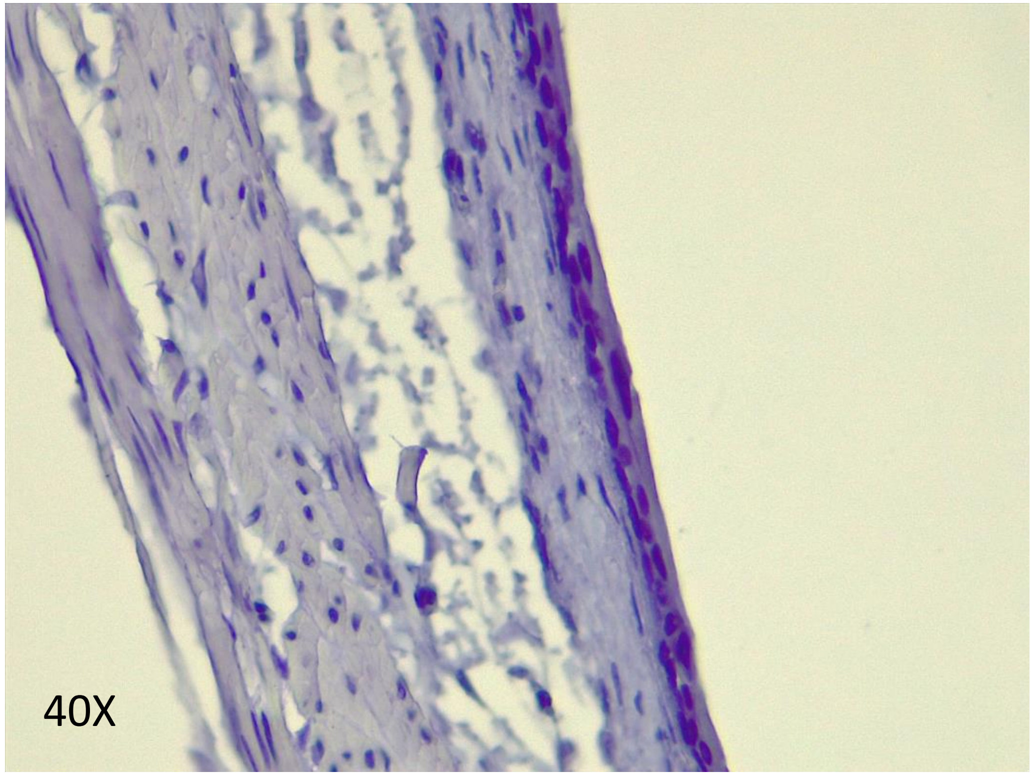

SA-HRP

Day 10 #2
CS

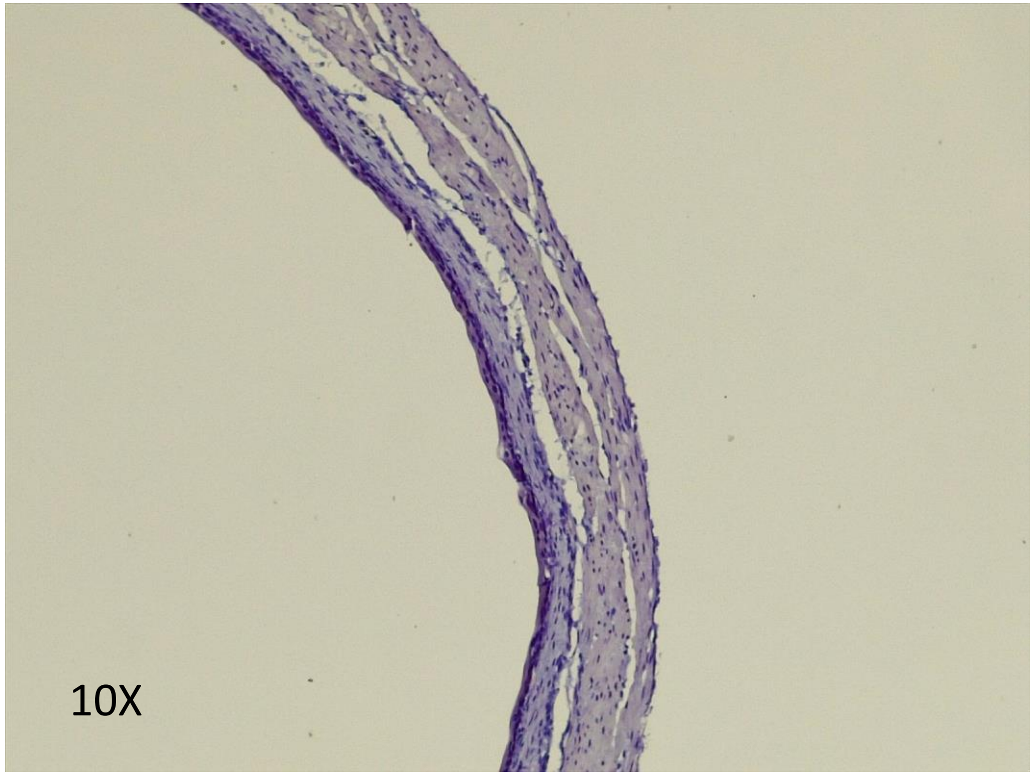

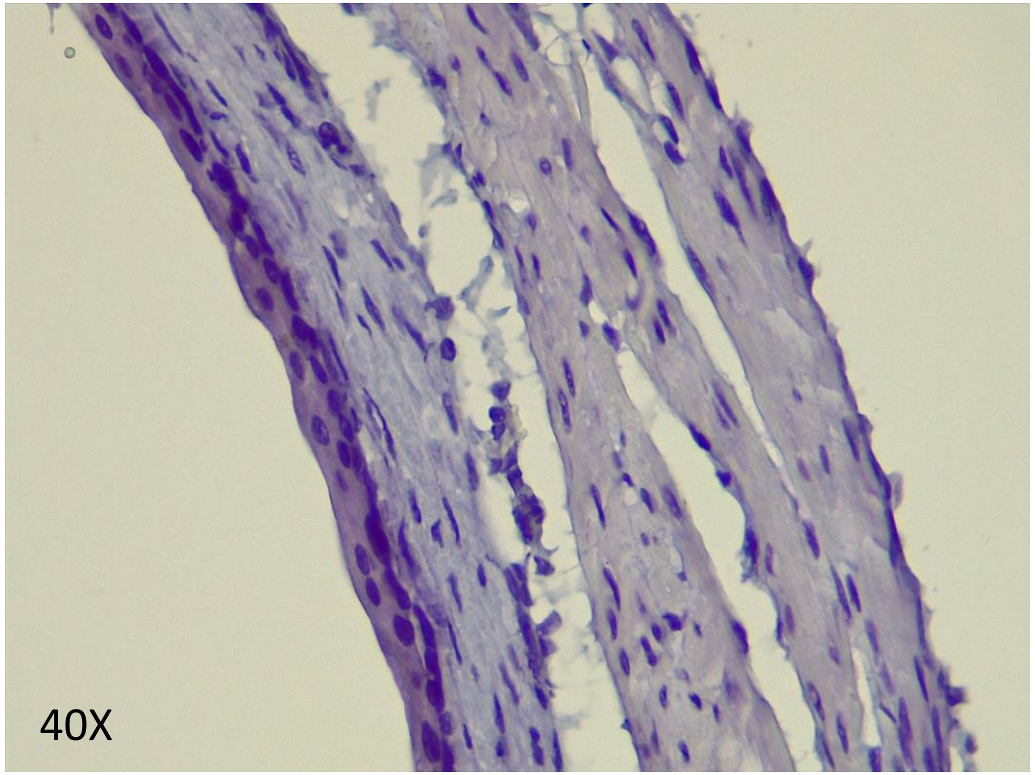

SA-HRP

Day 10 #3
CS

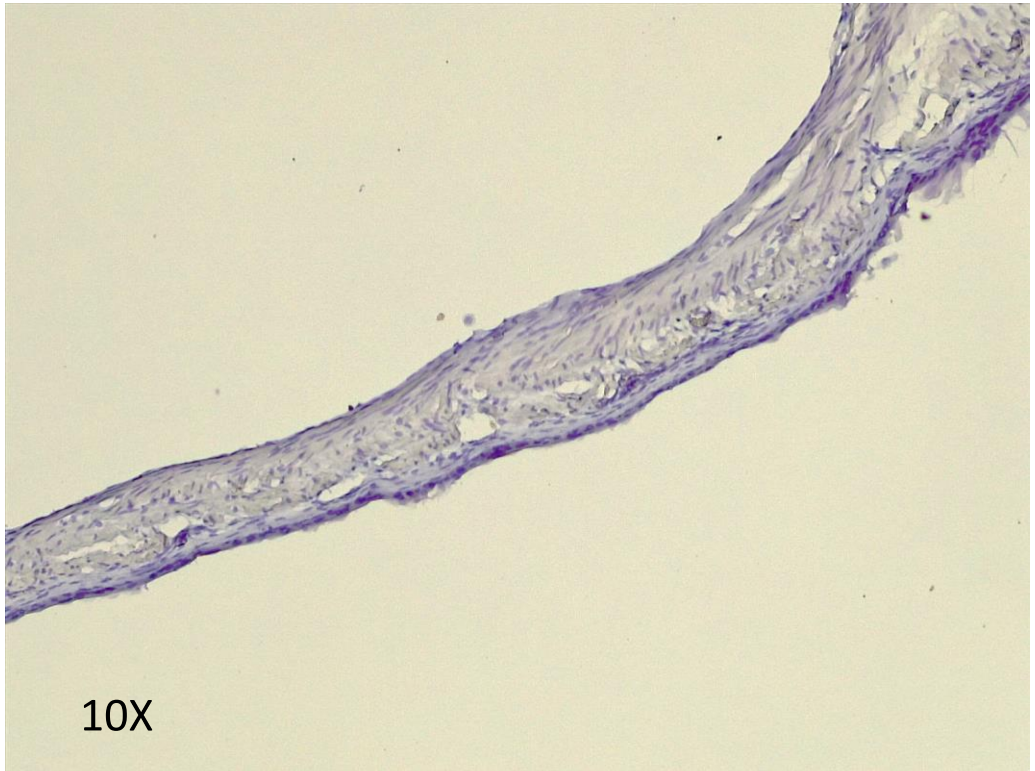

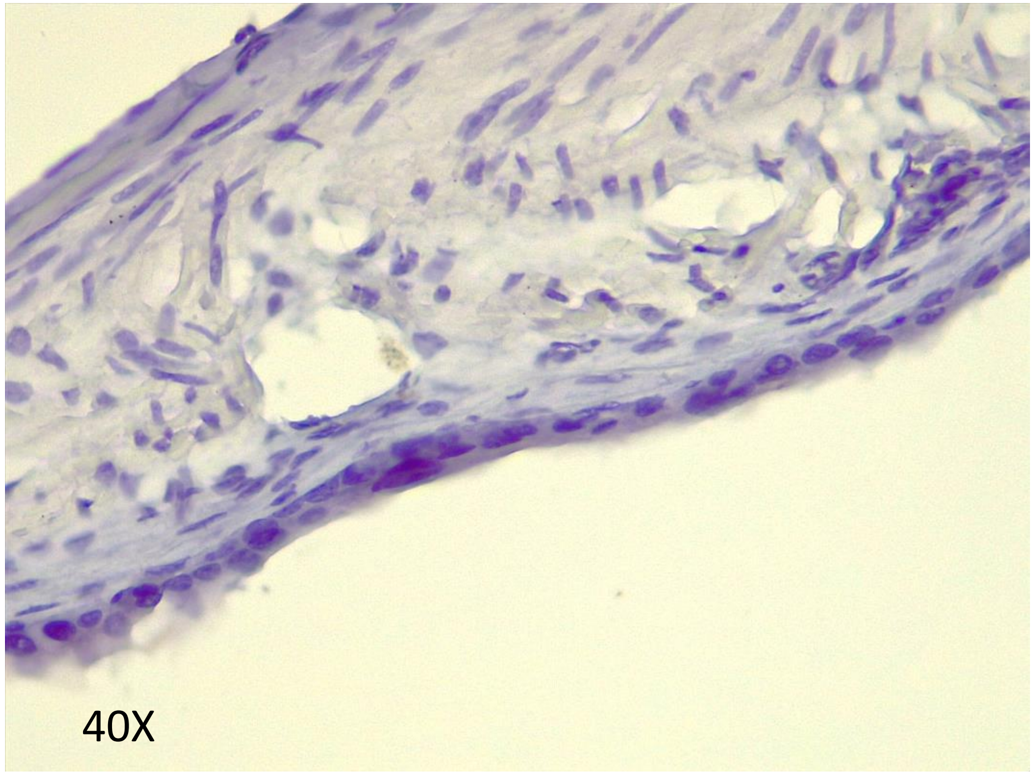

SA-HRP

Day 1 GAG

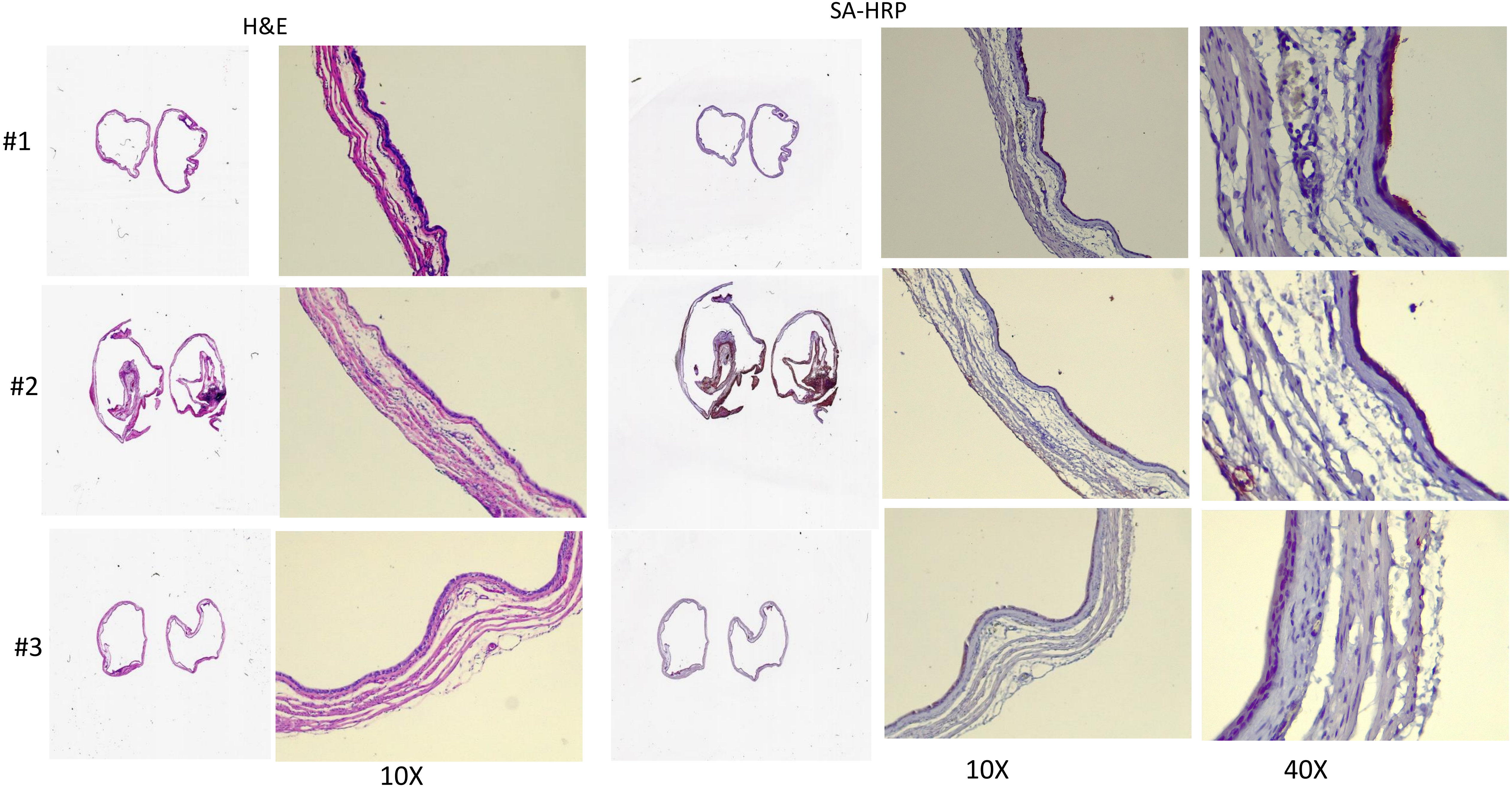

Day 1 #1
GAG

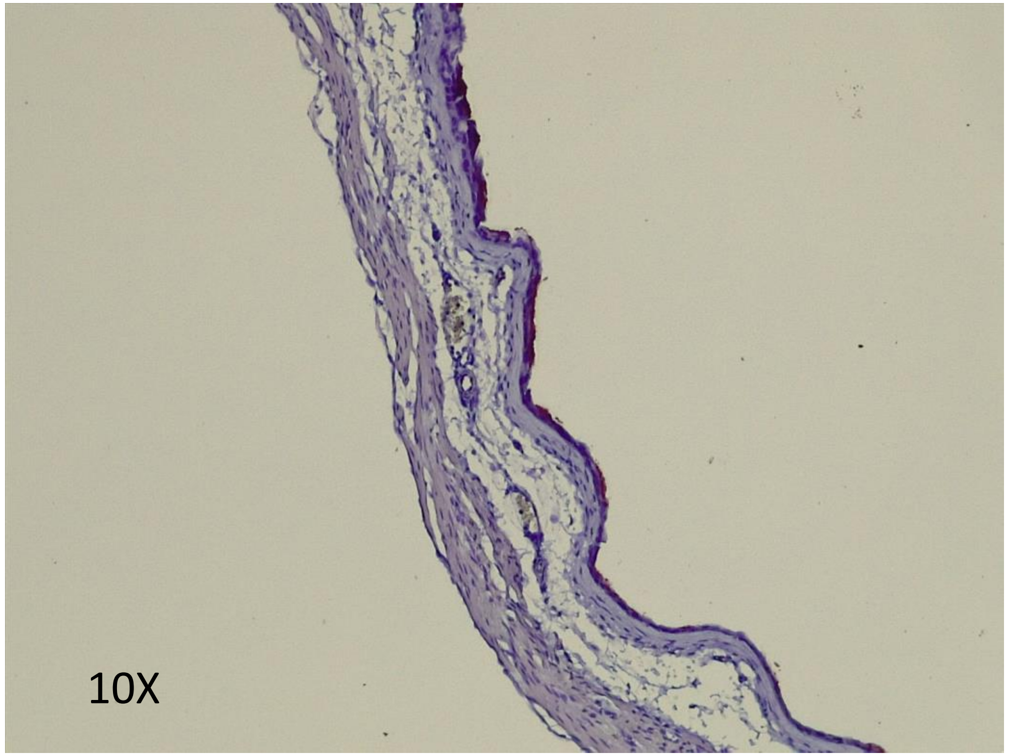

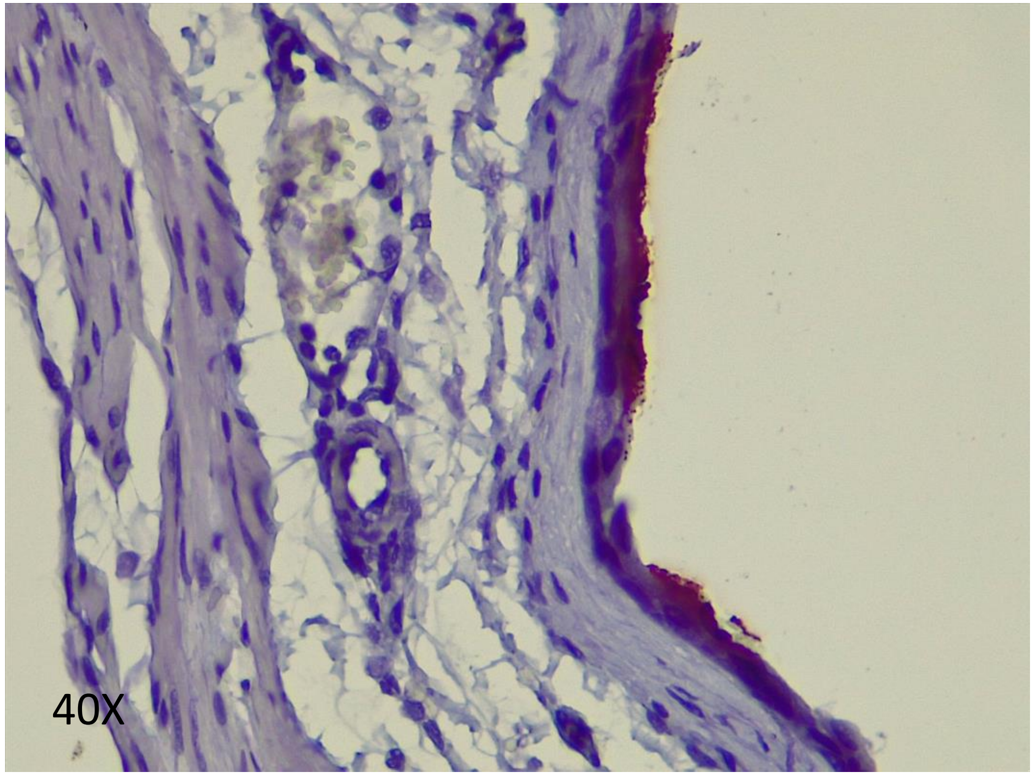

SA-HRP

Day 1 #2
GAG

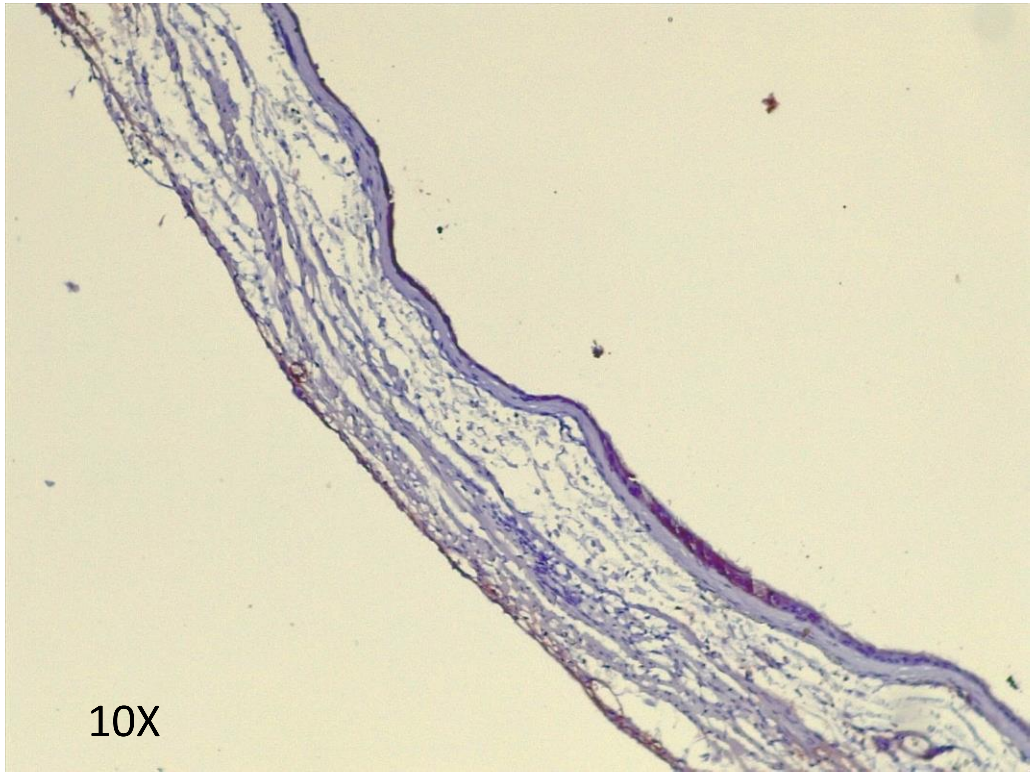

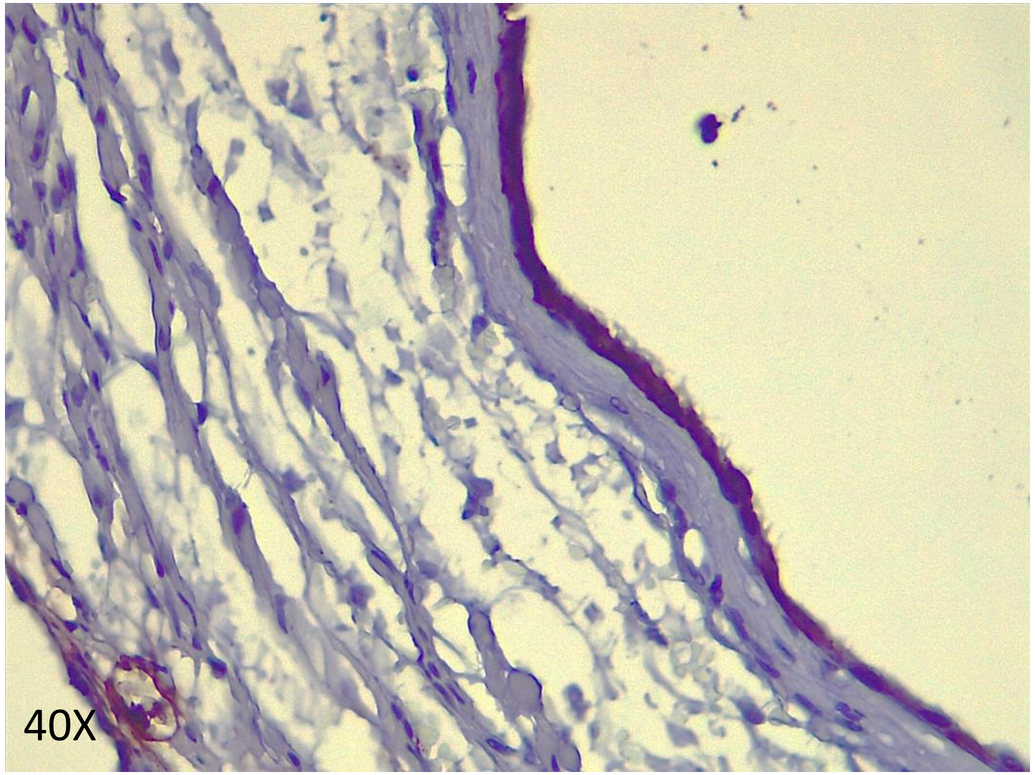

SA-HRP

Day 1 #3
GAG

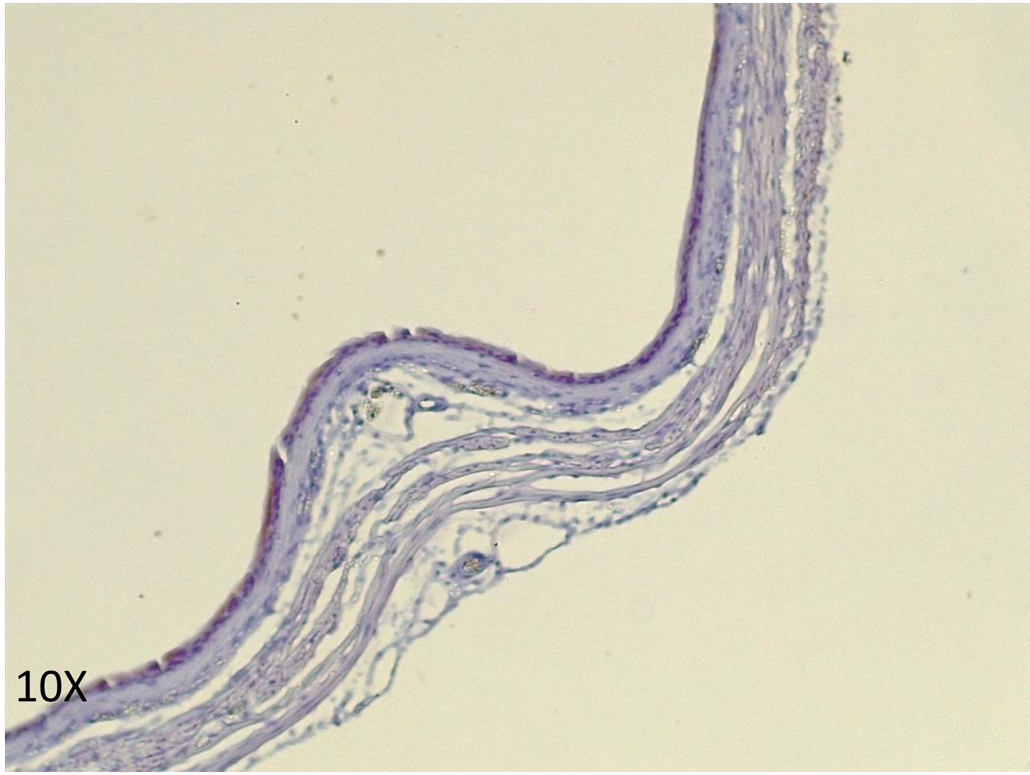

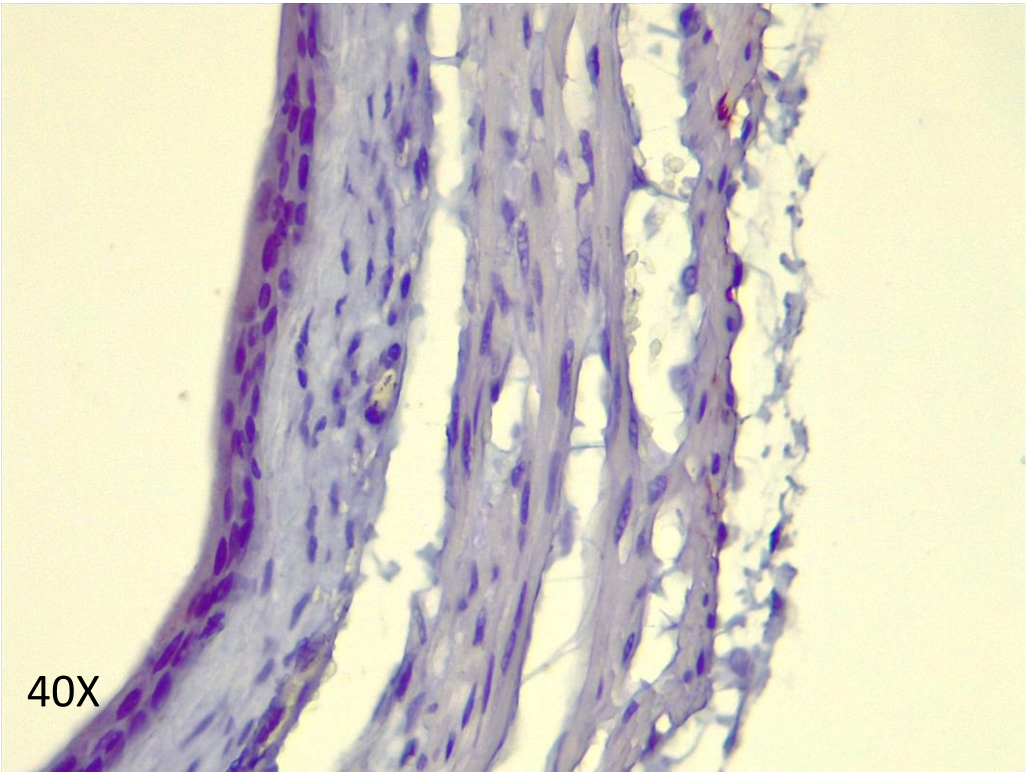

SA-HRP

Day 5 GLX-100

H&E

SA-HRP

#1

#2

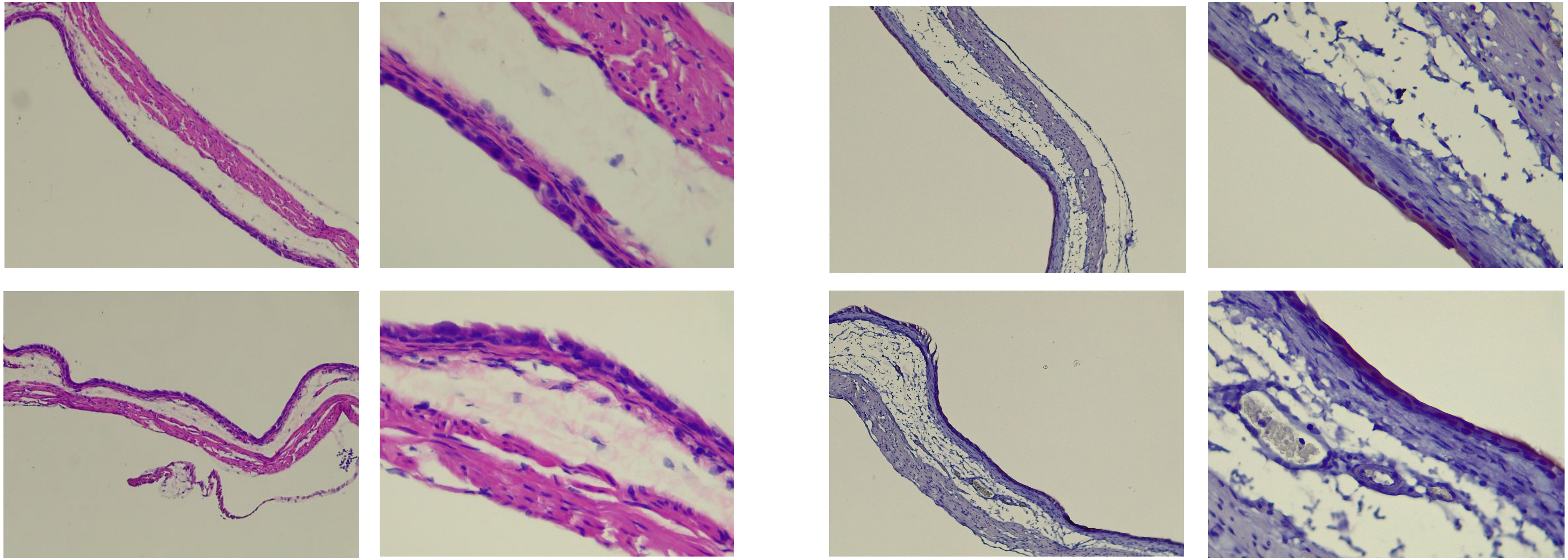

10X

40X

10X

40X

Day 5 #1

GLX-100

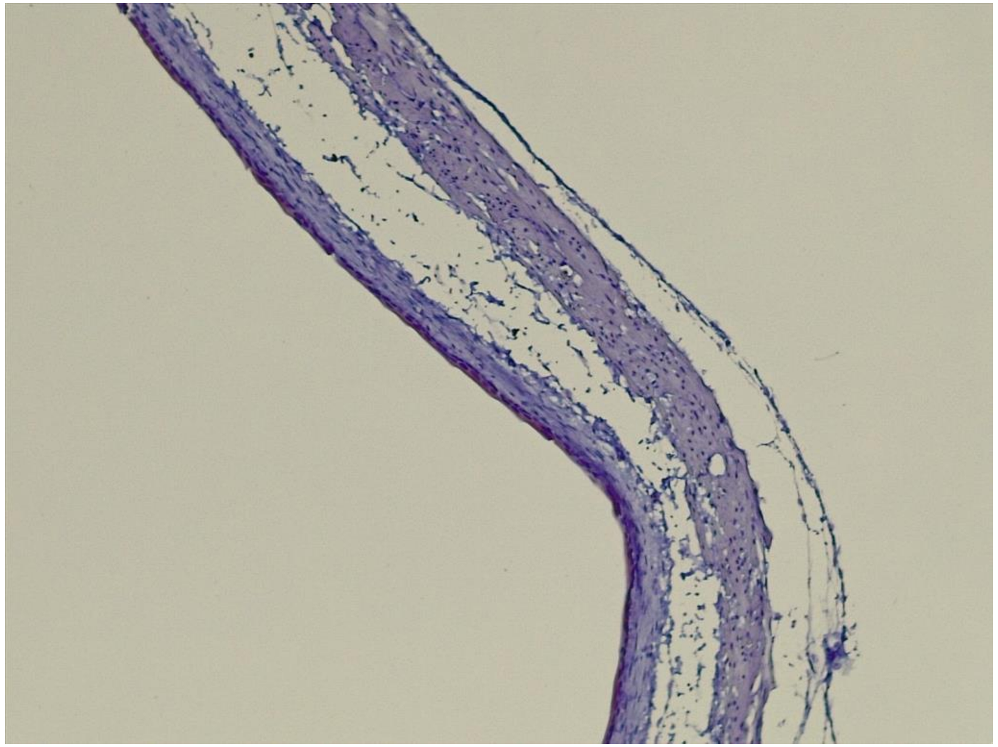

10X

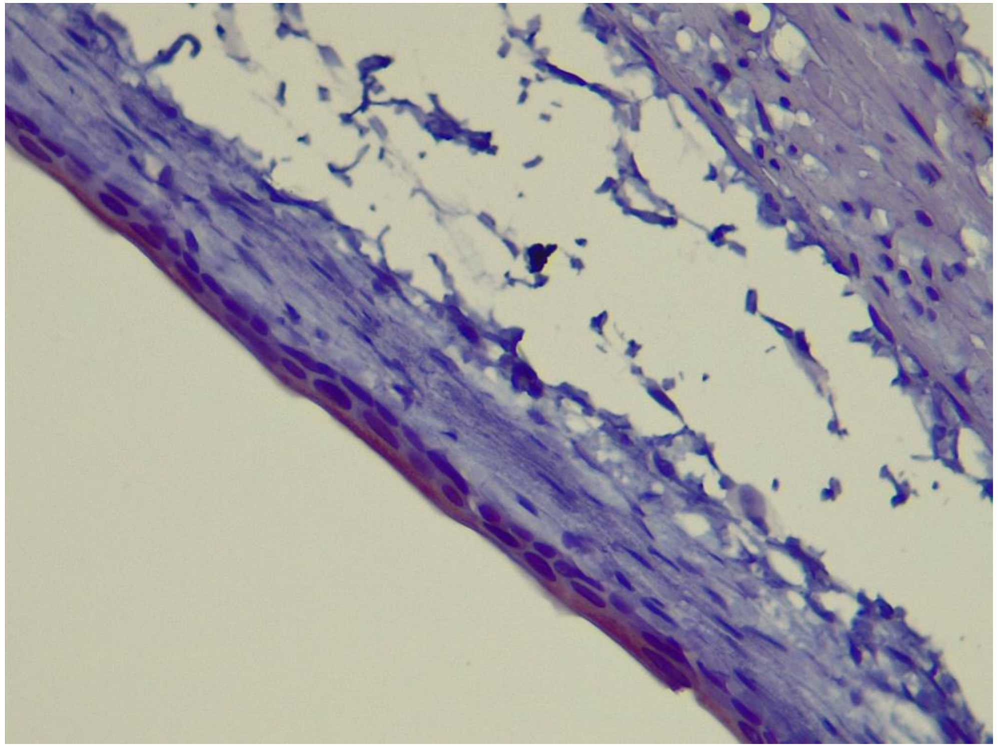

40X

SA-HRP

Day 5 #2

GLX-100

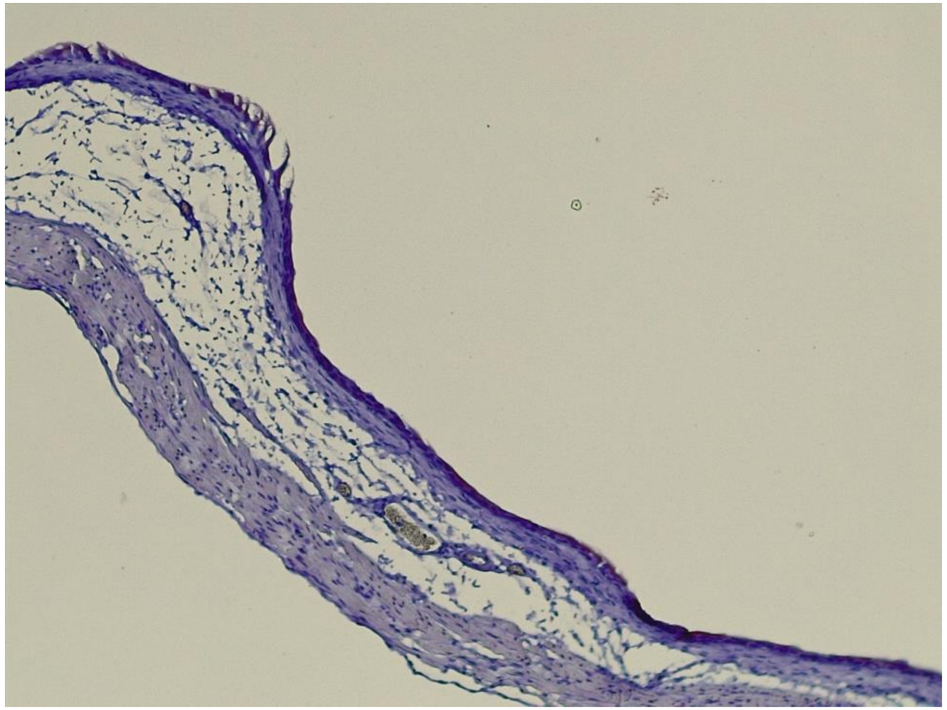

10X

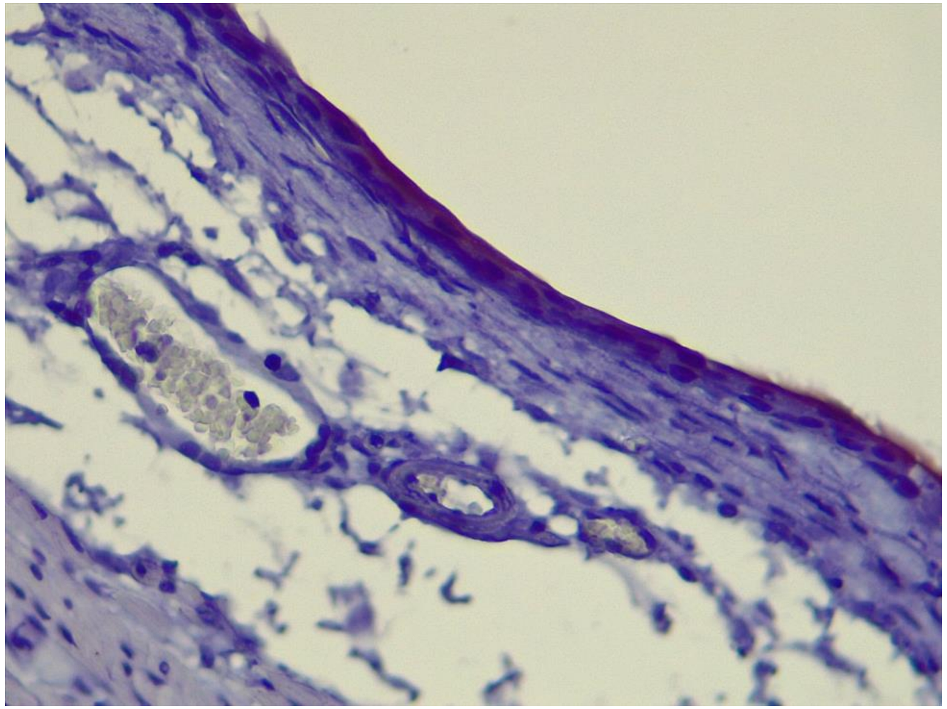

40X

SA-HRP

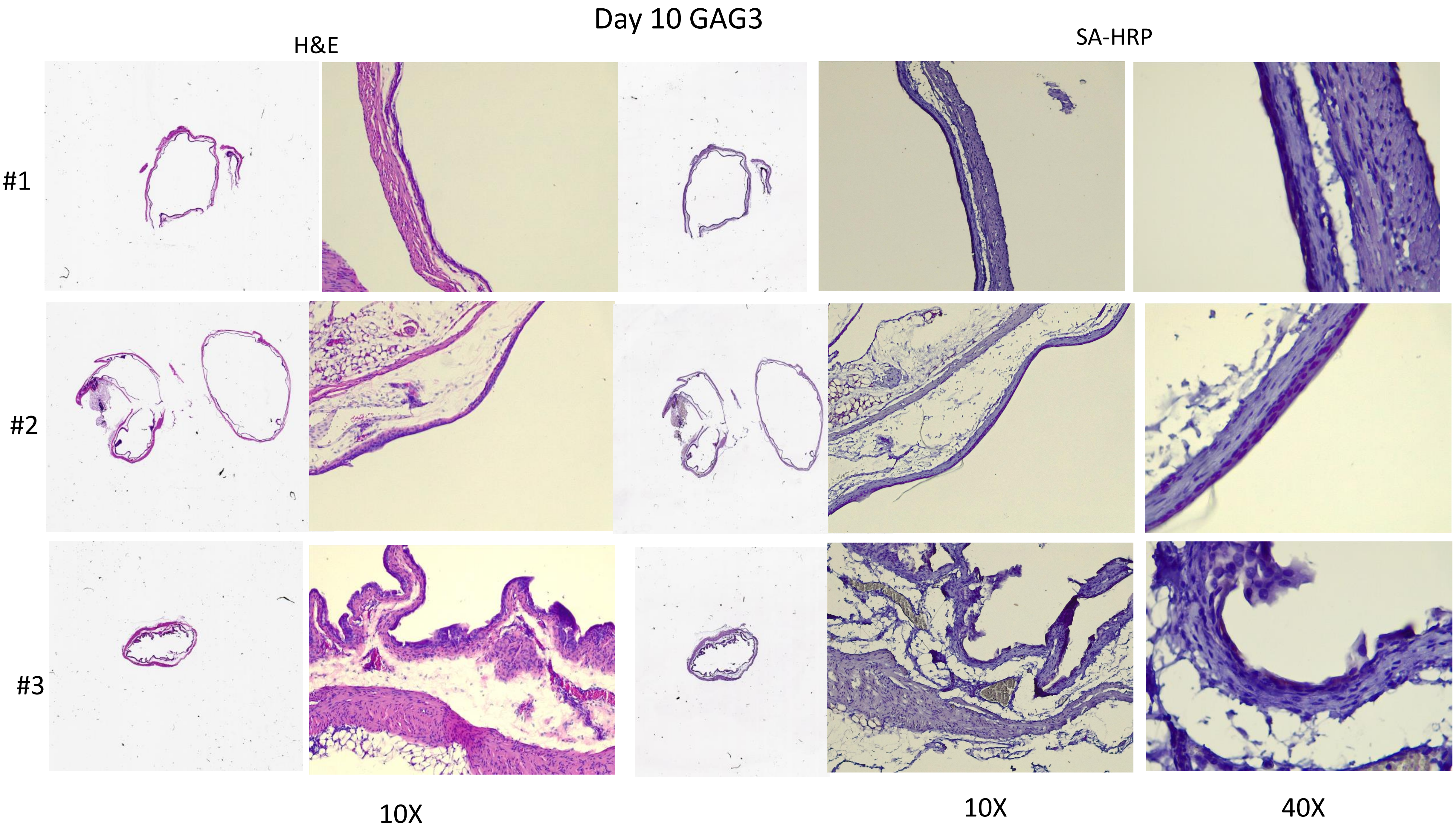
Day 10 GAG3
H&E
SA-HRP
#1
#2
#3
10X
10X
40X

Day 10 #1
GAG3

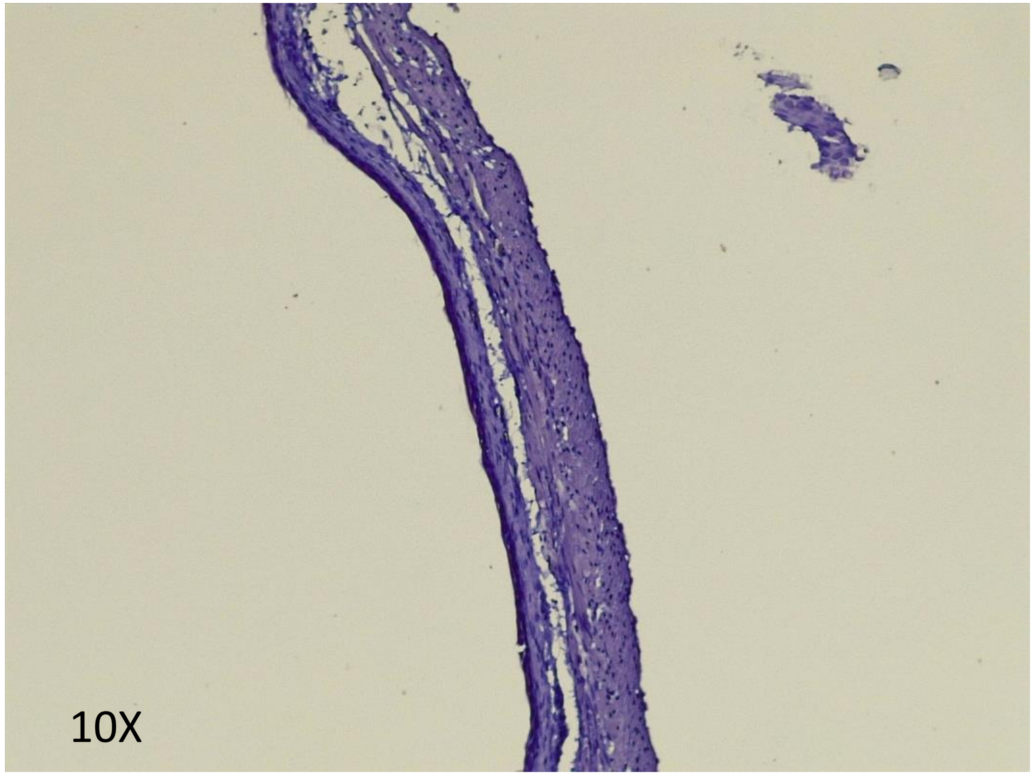

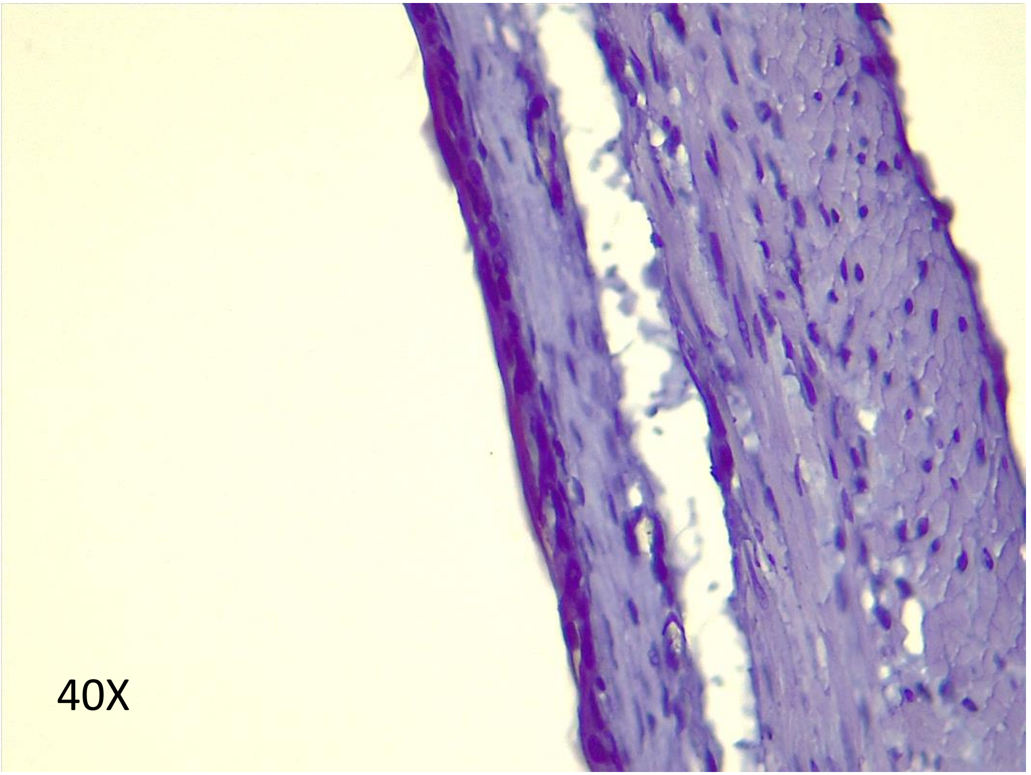

SA-HRP

Day 10 #2
GAG3

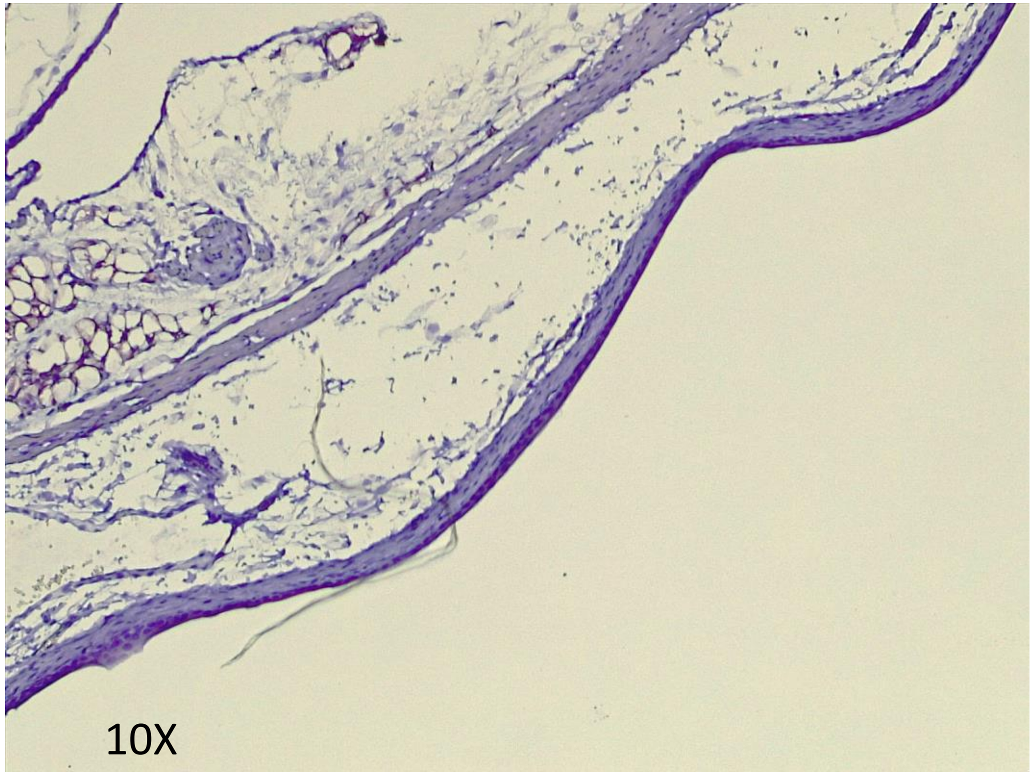

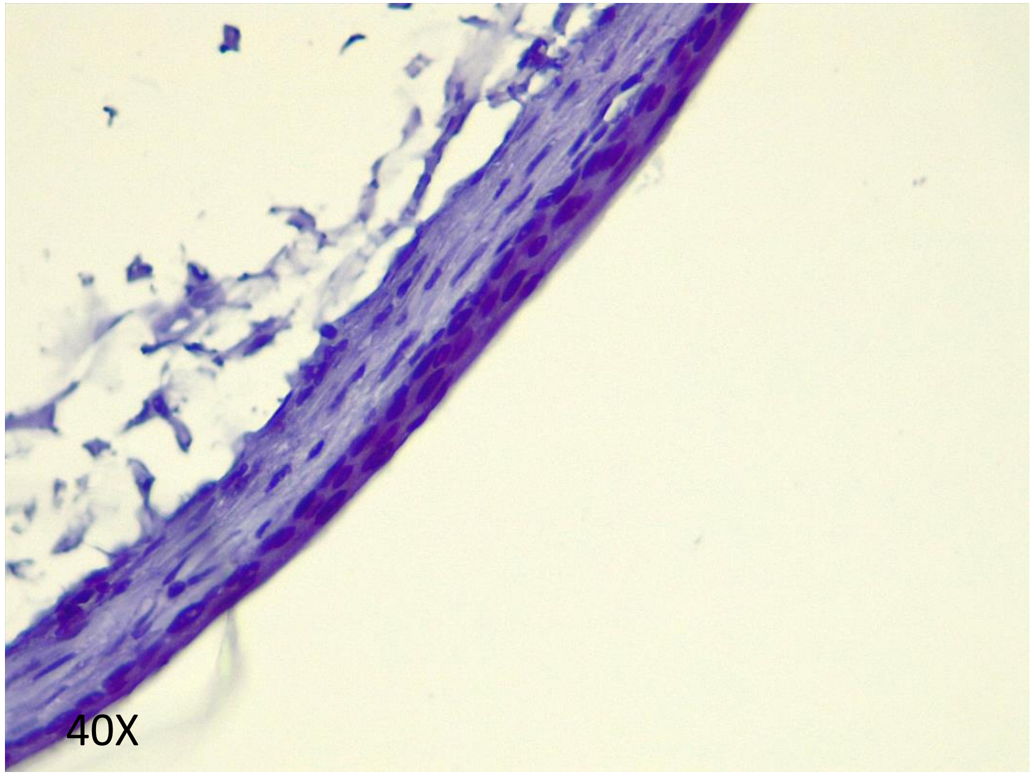

SA-HRP

Day 10 #3

GAG3

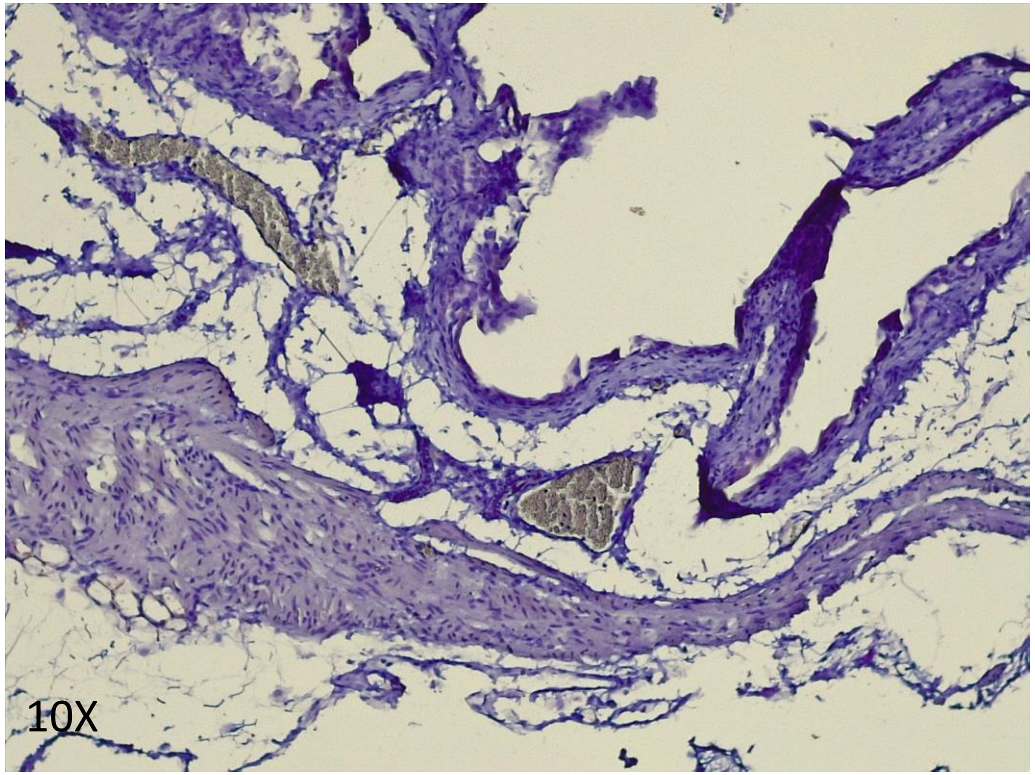

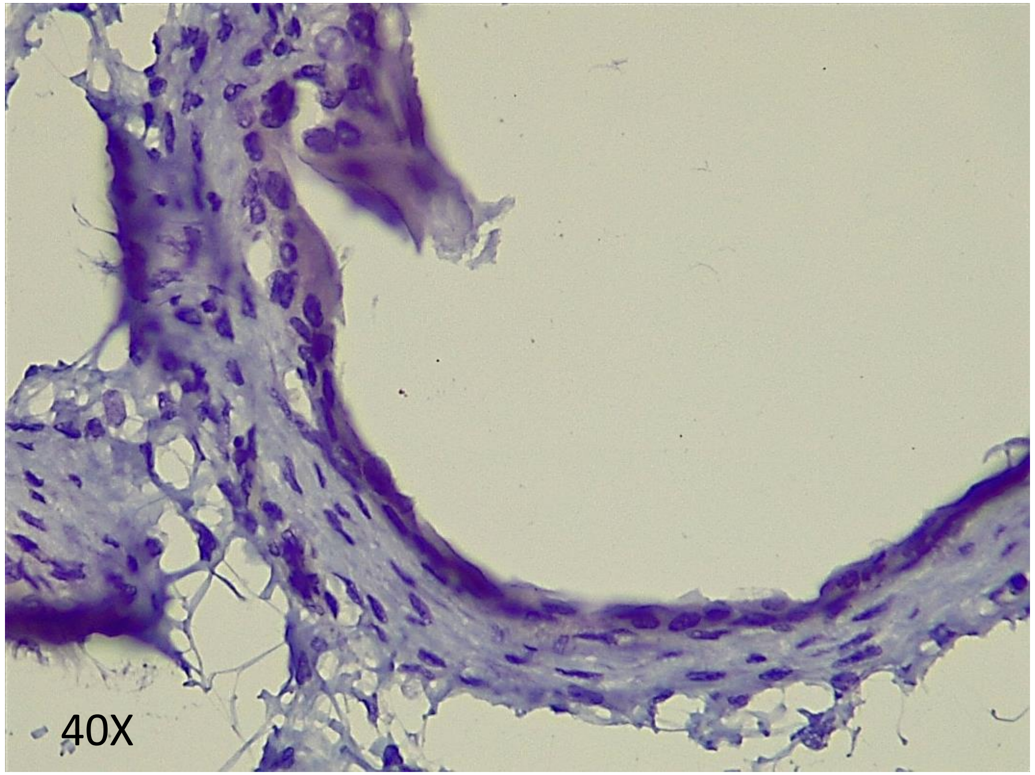

SA-HRP

GLX-100 –SA-HRP 40x

Day 1

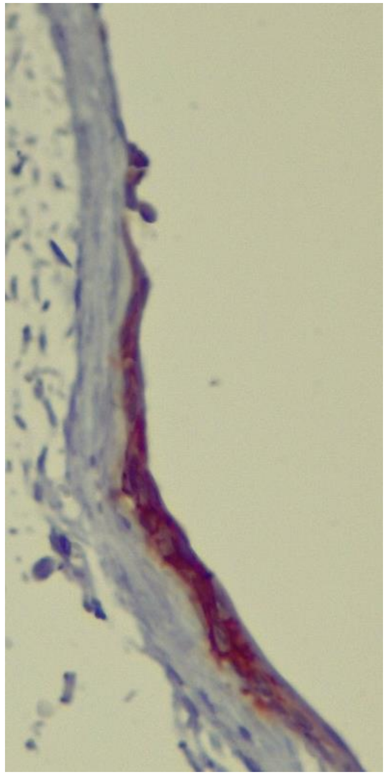

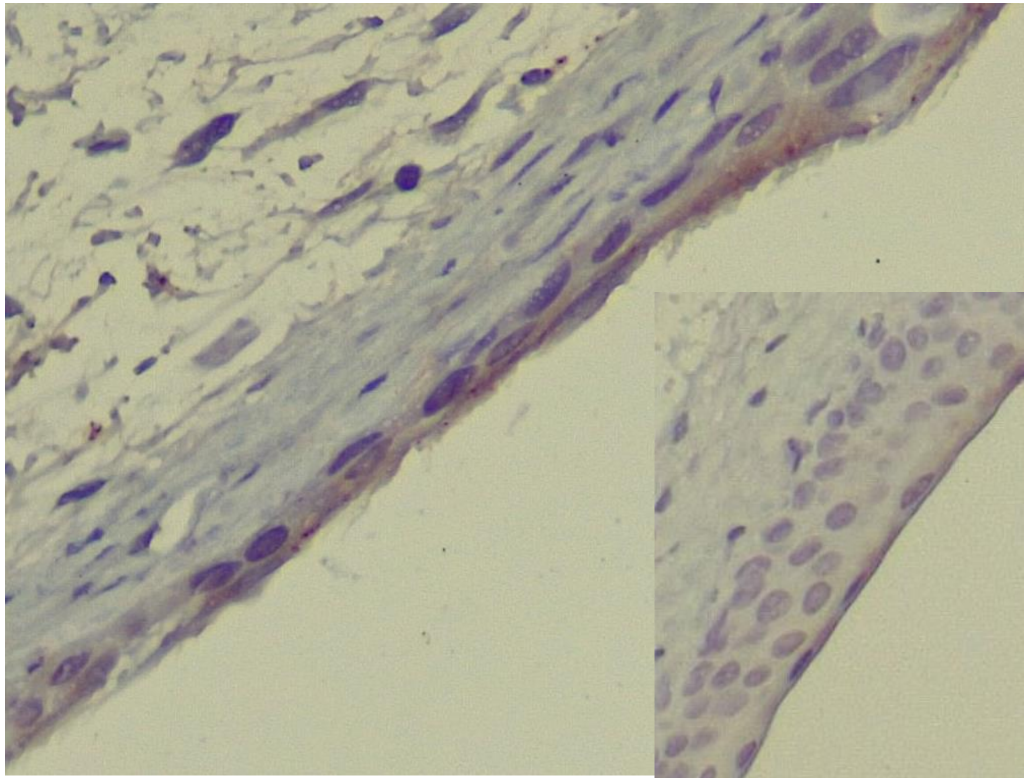

All mouse bladder walls have high levels of the GLX-100 1 day after administration

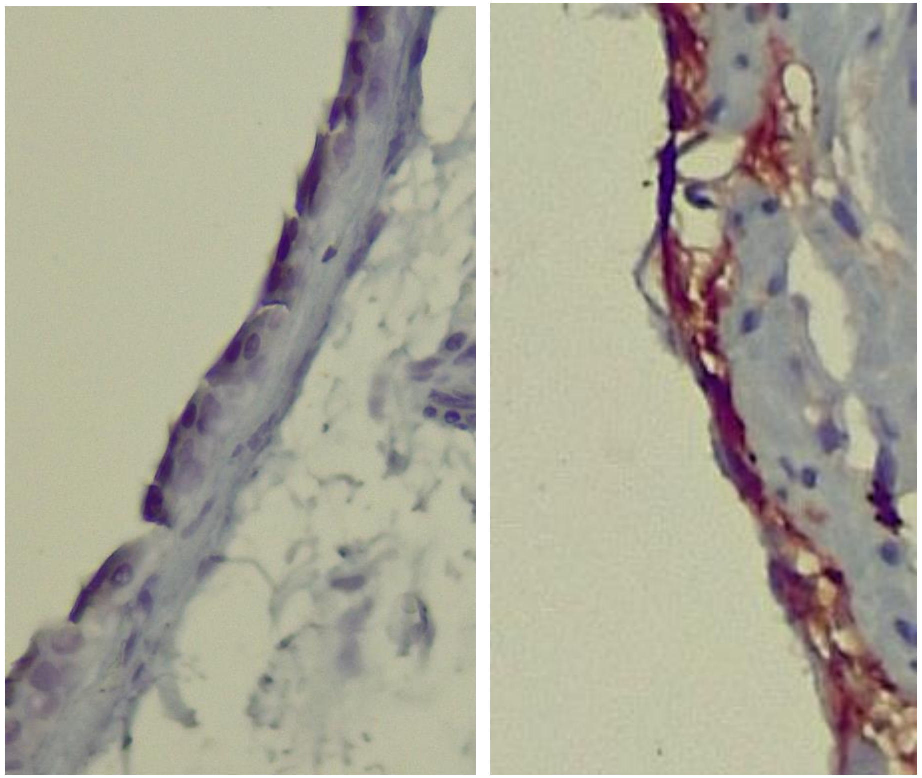

Control showing basal levels of uptake of SA-HRP

GLX-100 –SA-HRP 40x

Day 5

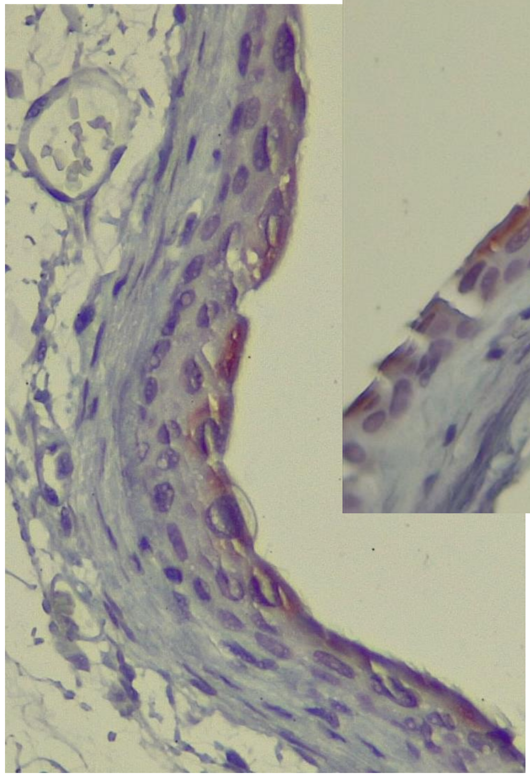

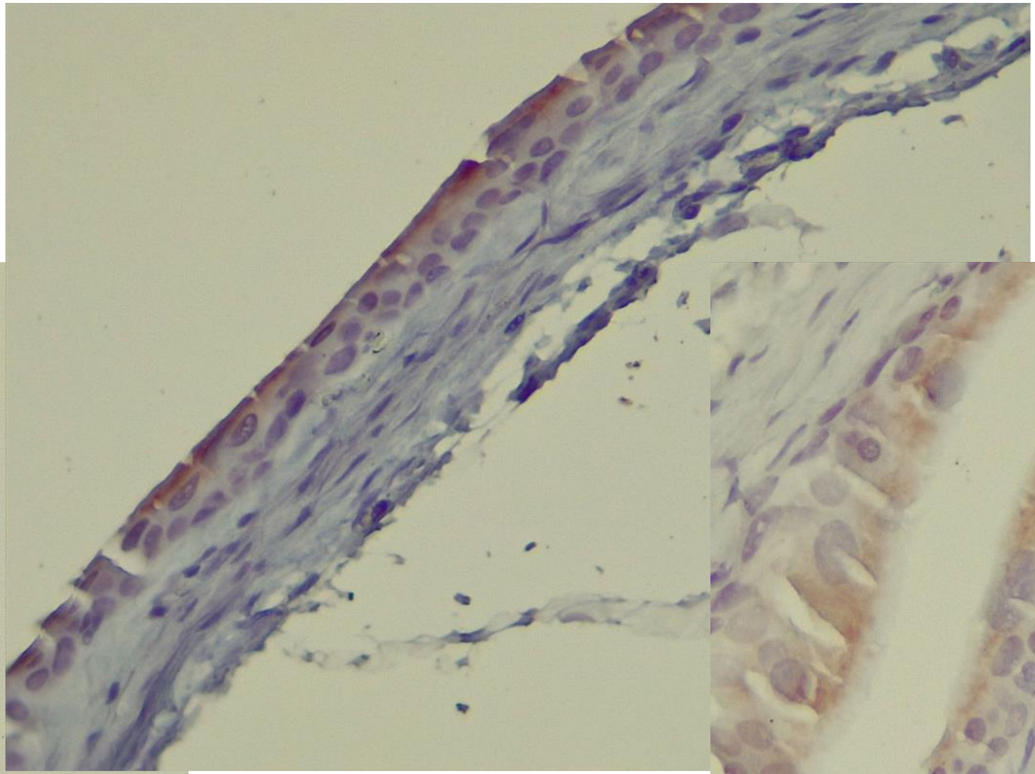

All mouse bladder walls still have fairly high levels of the GLX-100 5 days after administration

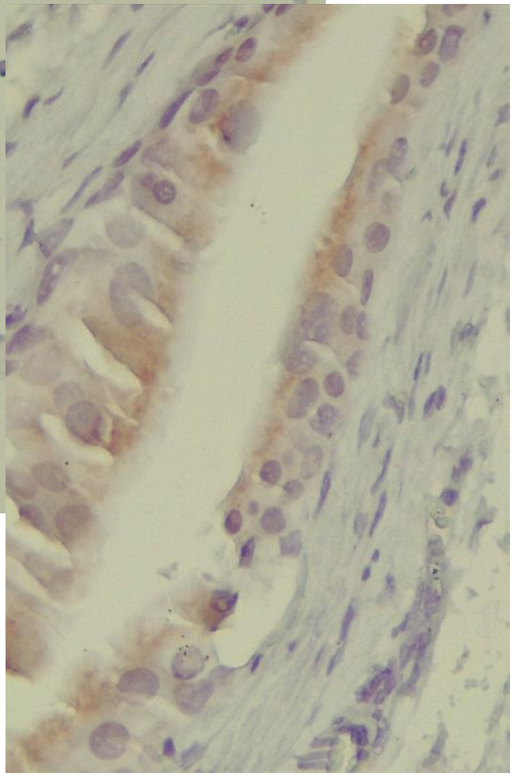

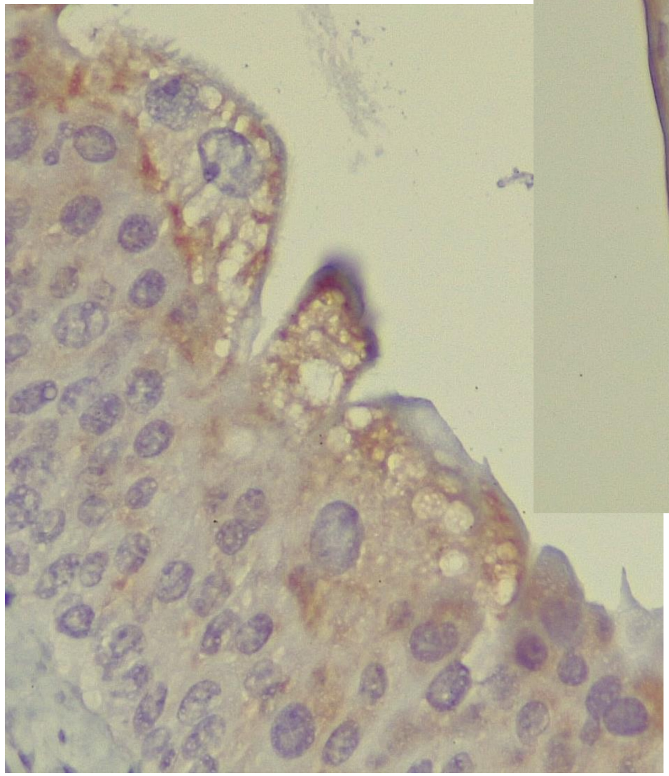

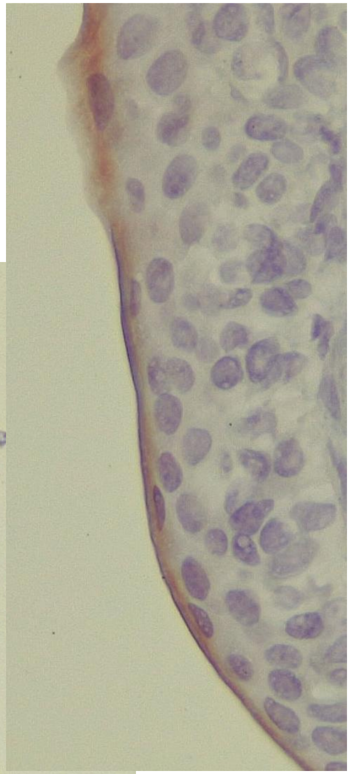

GLX-100 –SA-HRP 40x

Day 10

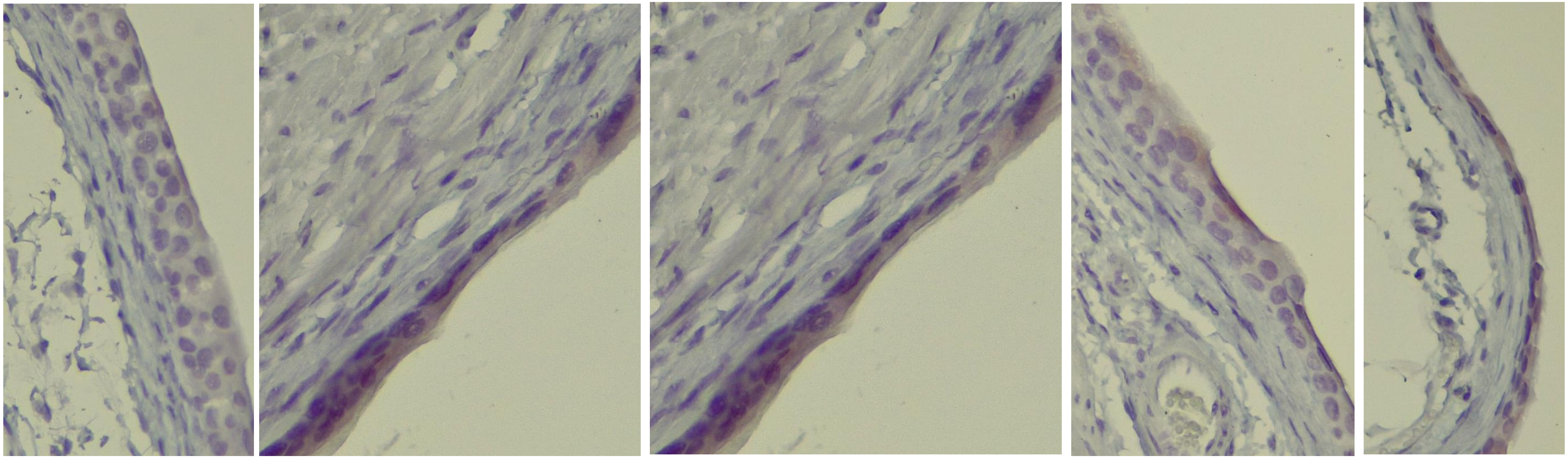

Most mouse bladder walls still have high levels of the GLX-100 10 days after administration

GLX-100 –SA-HRP

Day 1 Day 5 Day 10

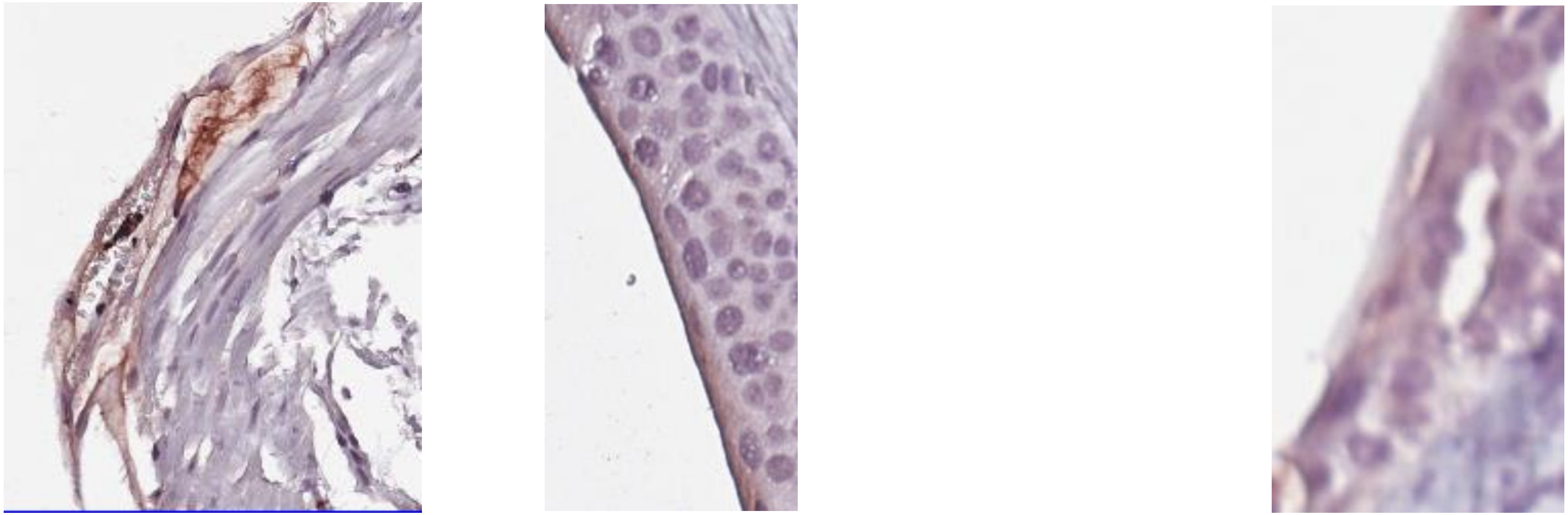

GLX-100
